# African swine fever virus DEAD-box helicase D1133L promotes OGG1-driven incision of genomic 8-oxoG via HDAC5 deacetylation

**DOI:** 10.1093/jmcb/mjaf029

**Published:** 2025-08-27

**Authors:** Jie Fan, Jifei Yang, Zhancheng Tian, Xiaoqiang Zhang, Shuxian Geng, Jianxun Luo, Istvan Boldogh, Qiaoying Zeng, Hong Yin, Guiquan Guan, Qingli Niu

**Affiliations:** State Key Laboratory for Animal Disease Control and Prevention, College of Veterinary Medicine, Lanzhou University, Lanzhou Veterinary Research Institute, Chinese Academy of Agricultural Sciences, Lanzhou 730000, China; African Swine Fever Regional Laboratory of China (Lanzhou), Gansu Province Research Center for Basic Disciplines of Pathogen Biology, Lanzhou 730046, China; College of Medicine, Northwest Minzu University, Lanzhou 730030, China; State Key Laboratory for Animal Disease Control and Prevention, College of Veterinary Medicine, Lanzhou University, Lanzhou Veterinary Research Institute, Chinese Academy of Agricultural Sciences, Lanzhou 730000, China; African Swine Fever Regional Laboratory of China (Lanzhou), Gansu Province Research Center for Basic Disciplines of Pathogen Biology, Lanzhou 730046, China; State Key Laboratory for Animal Disease Control and Prevention, College of Veterinary Medicine, Lanzhou University, Lanzhou Veterinary Research Institute, Chinese Academy of Agricultural Sciences, Lanzhou 730000, China; African Swine Fever Regional Laboratory of China (Lanzhou), Gansu Province Research Center for Basic Disciplines of Pathogen Biology, Lanzhou 730046, China; State Key Laboratory for Animal Disease Control and Prevention, College of Veterinary Medicine, Lanzhou University, Lanzhou Veterinary Research Institute, Chinese Academy of Agricultural Sciences, Lanzhou 730000, China; African Swine Fever Regional Laboratory of China (Lanzhou), Gansu Province Research Center for Basic Disciplines of Pathogen Biology, Lanzhou 730046, China; State Key Laboratory for Animal Disease Control and Prevention, College of Veterinary Medicine, Lanzhou University, Lanzhou Veterinary Research Institute, Chinese Academy of Agricultural Sciences, Lanzhou 730000, China; African Swine Fever Regional Laboratory of China (Lanzhou), Gansu Province Research Center for Basic Disciplines of Pathogen Biology, Lanzhou 730046, China; State Key Laboratory for Animal Disease Control and Prevention, College of Veterinary Medicine, Lanzhou University, Lanzhou Veterinary Research Institute, Chinese Academy of Agricultural Sciences, Lanzhou 730000, China; African Swine Fever Regional Laboratory of China (Lanzhou), Gansu Province Research Center for Basic Disciplines of Pathogen Biology, Lanzhou 730046, China; Department of Microbiology and Immunology, University of Texas Medical Branch at Galveston, Galveston, TX 77555, USA; College of Veterinary Medicine, Gansu Agricultural University, Lanzhou 730070, China; State Key Laboratory for Animal Disease Control and Prevention, College of Veterinary Medicine, Lanzhou University, Lanzhou Veterinary Research Institute, Chinese Academy of Agricultural Sciences, Lanzhou 730000, China; African Swine Fever Regional Laboratory of China (Lanzhou), Gansu Province Research Center for Basic Disciplines of Pathogen Biology, Lanzhou 730046, China; Jiangsu Co-Innovation Center for the Prevention and Control of Important Animal Infectious Disease and Zoonosis, Yangzhou University, Yangzhou 225009, China; State Key Laboratory for Animal Disease Control and Prevention, College of Veterinary Medicine, Lanzhou University, Lanzhou Veterinary Research Institute, Chinese Academy of Agricultural Sciences, Lanzhou 730000, China; African Swine Fever Regional Laboratory of China (Lanzhou), Gansu Province Research Center for Basic Disciplines of Pathogen Biology, Lanzhou 730046, China; State Key Laboratory for Animal Disease Control and Prevention, College of Veterinary Medicine, Lanzhou University, Lanzhou Veterinary Research Institute, Chinese Academy of Agricultural Sciences, Lanzhou 730000, China; African Swine Fever Regional Laboratory of China (Lanzhou), Gansu Province Research Center for Basic Disciplines of Pathogen Biology, Lanzhou 730046, China

**Keywords:** African swine fever virus, 8-oxoguanine DNA glycosylase 1, D1133L, acetylation, incision

## Abstract

African swine fever virus (ASFV) infection induces oxidative stress and produces oxidative DNA damage bases, leading to oxidative DNA base damage, including the formation of 8-oxoguanine (8-oxoG). Prompt repair of these lesions is essential to maintain genome stability. The enzyme 8-oxoguanine DNA glycosylase 1 (OGG1) initiates the base excision repair (BER) pathway by recognizing and incising 8-oxoG, while also regulating multiple biological processes through interactions with host and viral proteins. In this study, we identified a specific interaction between the N-terminal region of ASFV DEAD-box helicase D1133L and OGG1, establishing a unique role for ASFV D1133L in DNA BER. Furthermore, we demonstrated for the first time that ASFV D1133L is a substrate for the histone acetyltransferases CBP/p300 in the nucleus. Conversely, deacetylation of D1133L by HDAC5, which predominantly occurs in the cytoplasm through its interaction with OGG1, markedly enhances OGG1 incision activity on 8-oxoG. Taken together, our findings reveal a previously unrecognized function of ASFV D1133L in promoting 8-oxoG repair by binding to OGG1 to safeguard genome integrity.

## Introduction

African swine fever virus (ASFV) is a double-stranded DNA virus, which is the only member of the *Asfaviridae* family (Wang et al., 2019; Gaudreault et al., 2020). ASFV encodes >150 proteins to ensure successful invasion, replication, transcription, and the evasion of host immune response (Wang et al., 2021). RNA helicases are important for viral transcription and translation (Freitas et al., 2019; Ramirez-Medina et al., 2021). Previous study indicated that ASFV C962R protein containing helicase domain likely works on the forked DNA in DNA base excision repair (BER) pathway (Shao et al., 2023). Similarly, ASFV-encoded D1133L, containing a highly conserved ‘Asp–Glu–Ala–Asp’ (DEAD) motif, has been predicted to be a DEAD-box helicase belonging to superfamily II (SFII) and involved in ASFV transcriptional initiation (Hao et al., 2022). However, the physiological significance of D1133L in ASFV infection has not been elucidated.

Potentially, the biological function of ASFV D1133L is similar to that of other SFII helicases to a large extent. SFII contains diverse helicase families, including type I restriction enzymes and RecQ-like, RecG-like, Rad3/XPD, Ski2-like, RIG-I-like, NS3/NPH-II, Swi/Snf, DEAH/RHA, and DEAD-box families (Hodroj et al., 2017). These helicases are involved in a wide variety of cellular processes of DNA and RNA metabolism, such as replication, transcription, translation, chromatin rearrangement, nuclear export, ribosome biogenesis, RNA maturation, and DNA repair (Tanner and Linder, 2001; Owttrim, 2006; Tabassum and Ghosh, 2023). The DEAD-box helicase family is most conserved in SFII and mainly functions to promote genomic stability through preventing DNA damage, participating directly in the repair of DNA damage, and regulating the expression of related factors (Chan et al., 2014; Dutertre et al., 2014). A growing number of studies have shown that DEAD-box helicases are localized to DNA damage sites by binding to damage-repairing proteins. For instance, DEAD box 1 (DDX1) was identified rapidly accumulating at irradiation-induced foci and co-localized with γ-H2AX in double-strand breaks (DSBs) (Li et al., 2008), followed by the interaction with ATM protein and phosphorylation (Polo and Jackson, 2011; Osipov et al., 2023). DDX3X is recruited to DNA damage sites in a poly(ADP-ribose) polymerase 1 (PARP1)-dependent manner (Cargill et al., 2021b). DDX5 is an active participant in DSBs and interacts with Ku (Abbasi and Schild-Poulter, 2019). Depletion of DDX19 causes the accumulation of DNA breaks, substantiating the role of helicases in genomic stability (Hodroj et al., 2017). Other DEAD-box helicases, such as DDX21, DDX39B, and DDX41, are also involved in maintaining genomic stability by regulating DNA damage repair (Cargill et al., 2021a). However, the role of DEAD-box helicases in oxidative DNA damage repair has not been clarified.

The oxidative damage marker 8-oxoguanine (8-oxoG) is primarily repaired by BER, which is initiated by 8-oxoguanine DNA glycosylase 1 (OGG1) (Fouquerel et al., 2019). Our previous study showed that ASFV infection induces oxidative DNA damage, and OGG1 plays a pivotal role in its pathogenesis (Fan et al., 2023). Moreover, a recent study revealed that the helicase RecQ-like helicase 4 (RECQL4) is involved in 8-oxoG repair by binding to OGG1 and SIRT1 indirectly modulates OGG1 activity through maintaining RECQL4 in a hypoacetylated state (Duan et al., 2020). Thus, we asked whether ASFV DEAD-box helicase D1133L is involved in BER by interacting with OGG1. Intriguingly, we previously found D1133L interaction with OGG1 by co-immunoprecipitation (co-IP) and mass spectrometry (MS) (data not shown). Here, we show that D1133L participates in the elimination of 8-oxoG under oxidative stress and ASFV infection.

As previously shown, the function of helicases can be regulated by acetylation and deacetylation. Dna2 nuclease/helicase is acetylated by p300, stimulating its 5′–3′ helicase and DNA-dependent ATPase activities (Balakrishnan et al., 2010). DDX5 and DDX17 are substrates for the acetyltransferase p300, and the acetylation increases their ability to co-activate estrogen receptor (Mooney et al., 2010). Moreover, DDX3X becomes acetylated upon stress but deacetylated by HDAC6 for the formation of stress granules (Saito et al., 2021). Our data in this study provide novel mechanistic insights into the role of acetylation in regulating D1133L activity, showing that acetylation by CBP/p300 inhibits D1133L activity, while deacetylation by HDAC5 enhances the activity of D1133L to promote OGG1-driven 8-oxoG incision. These results reveal a previously uncharacterized post-transcriptional regulatory mechanism of D1133L acting as a regulator of DNA BER, binding to OGG1, thereby contributing to the safeguard of genome integrity.

## Results

### ASFV helicase D1133L interacts with OGG1

**Figure 1:**
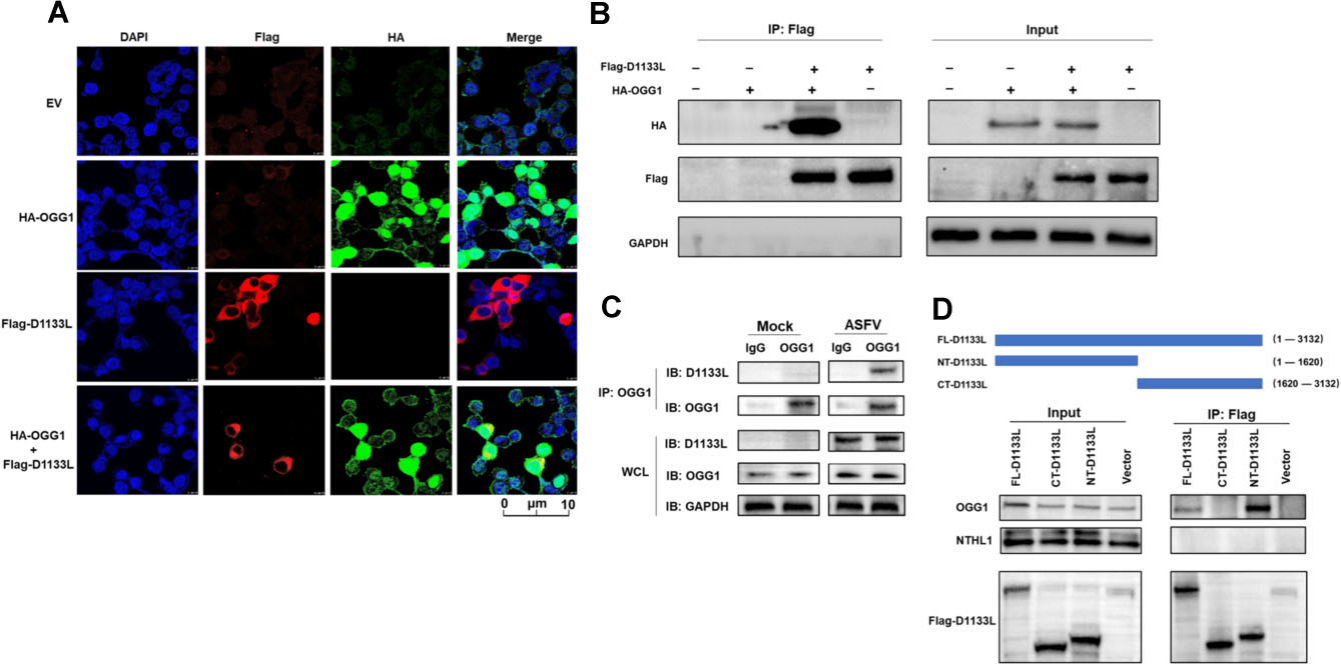
D1133L interacts with OGG1. (**A**) Subcellular localization of D1133L and OGG1. HEK 293T cells were transfected with Flag-D1133L (2 μg) and/or HA-OGG1 (2 μg) for 24 h. Confocal microscopy was employed to detect subcellular localization, using anti-Flag and anti-HA antibodies. Yellow fluorescence indicates co-localization, while DAPI stains the nucleus. Scale bar, 10 μm. (**B**) Interaction between OGG1 and D1133L. HEK 293T cells were transfected with HA-OGG1 (2 μg) and/or Flag-D1133L (2 μg) for 24 h and subjected to Flag IP, followed by immunoblotting (IB) using anti-HA or/and anti-Flag antibodies. (**C**) OGG1 and D1133L interaction during ASFV infection. PAMs were infected with or without ASFV (MOI = 1) for 24 h and subjected to OGG1 IP, followed by IB using anti-OGG1 and anti-D1133L antibodies. (**D**) NT-D1133L interacts with OGG1 in MA104 cells. MA104 cells were transfected with different domains of D1133L (FL-D1133L, NT-D1133L, and CT-D1133L) tagged with 3×Flag (2 μg each) for 24 h and subjected to Flag IP, followed by IB using anti-Flag, anti-OGG1, or anti-NTHL1 antibodies. WCL, whole-cell lysate.

Our previous MS data revealed that ASFV-D1133L was an OGG1-associated protein (data not shown). To further confirm the interaction between D1133L and OGG1, we performed a confocal immunofluorescence assay. The results showed that D1133L and OGG1 were predominantly localized in the cytoplasm of HEK 293T cells (Figure 1A). Meanwhile, co-IP analysis indicated that OGG1 interacted with D1133L both *in vitro* and during ASFV infection (Figure 1B and C). Subsequently, we explored the domain(s) of D1133L involved in the interaction with OGG1. Based on the functional regions of D1133L, i.e. an N-terminal DEAD-box helicase domain and a C-terminal ATP tail, we generated two truncated forms of D1133L and assessed their co-precipitation with OGG1 (Figure 1D). The N-terminal domain of D1133L (NT-D1133L) exhibited a slightly stronger affinity for OGG1 compared with full-length D1133L (FL-D1133L), while deletion of the N-terminal domain (CT-D1133L) resulted in the loss of binding with OGG1 (Figure 1D; Supplementary Figure S1). These results implied that the N-terminal DEAD-box domain of D1133L plays a key role in OGG1 binding, while the C-terminal domain is largely dispensable.

### D1133L is participated in OGG1-driven 8-oxoG elimination

**Figure 2:**
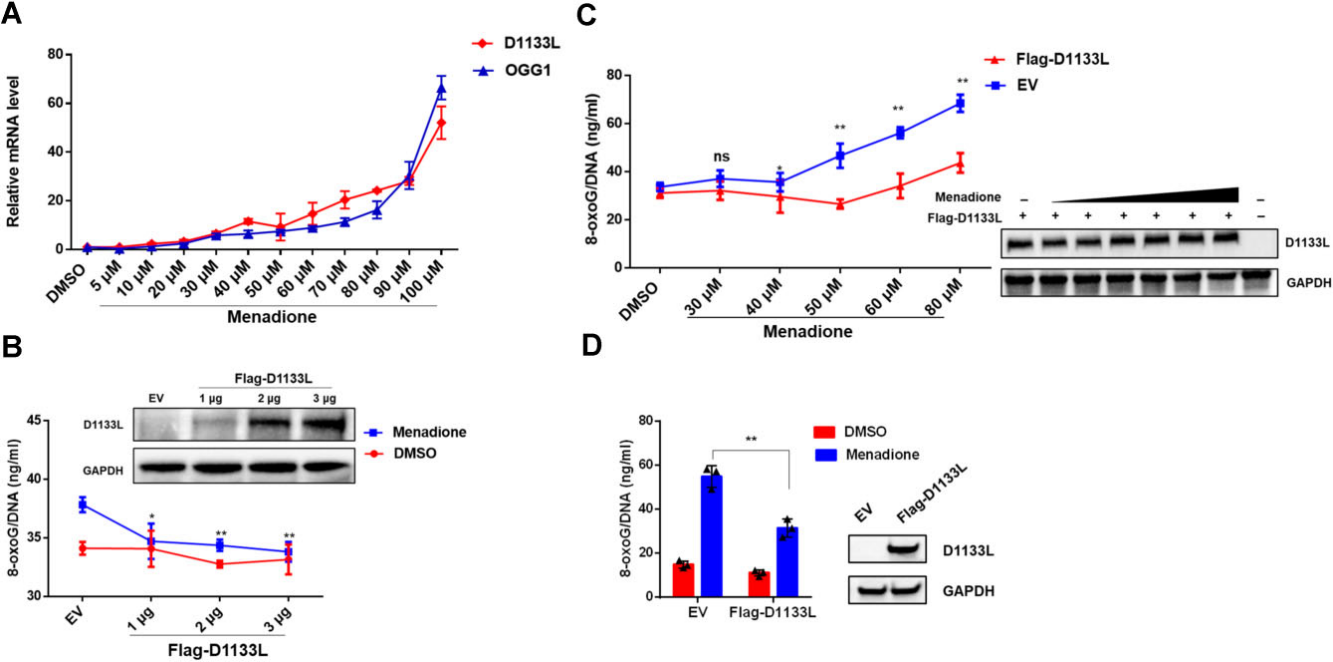
D1133L is required for 8-oxoG elimination. (**A**) The mRNA level of D1133L and OGG1 under oxidative stress. PAMs were treated with specified concentrations of menadione for 3 h before ASFV infection. Samples were collected at 24 h post ASFV infection and analyzed by RT-qPCR for the mRNA levels of D1133L and OGG1. Data are presented as mean ± SD from three independent experiments. (**B** and **C**) Increased genomic 8-oxoG levels in Flag-D1133L-expressing and menadione-treated cells. HEK 293T cells were transfected with Flag-D1133L (0, 1, 2, 3 μg) for 24 h and then treated with menadione (40 μM) or DMSO for 6 h (**B**) or transfected with Flag-D1133L (2 μg) or empty vector (EV) for 24 h and then treated with increasing concentrations of menadione for 6 h (**C**). 8-oxoG levels were assessed by ELISA. Western blot analysis of Flag-D1133L expression is shown. Data are presented as mean ± SD (*n* = 3). Statistical significance was performed using Student's *t*-test, compared to EV. **P < 0.01; *P < 0.05; ns, not significant. (**D**) The change of 8-oxoG levels by D1133L in the presence of menadione. HEK 293T cells cultured in DMEM medium with or without 40 μM menadione were transfected with Flag-D1133L (2 μg) for 24 h, and then 8-oxoG levels were measured by ELISA. Western blot analysis confirmed the overexpression of Flag-D1133L. Data are presented as mean ± SD (*n* = 3). Statistical analysis was performed using Student's *t*-test. **P < 0.01.

Oxidative stress is the most common inducer that leads to oxidative DNA base damage. The DNA repair enzyme OGG1, which is expressed in both the nucleus and cytoplasm, initiates the repair of oxidative base lesions in nuclear and mitochondrial DNA, such as 8-oxoG, during BER (Jun et al., 2022). Previous studies have shown the increased expression of OGG1 in ASFV-infected cells, suggesting the involvement of OGG1 in repairing oxidative damage to the viral genome (Fan et al., 2023). D1133L was found to express in the late phase of infection (Supplementary Figure S2). To explore the potential link between D1133L and oxidative stress, we used menadione, which generates reactive oxygen species (ROS) via redox cycling, to increase the level of oxidative stress in cells (Szeliga and Rola, 2022). We found that menadione at non-toxic doses, determined with cell viability assays (Supplementary Figure S3A), increased both OGG1 and D1133L mRNA levels in the primary porcine alveolar macrophages (PAMs) (Figure 2A). ELISA results indicated that menadione-induced genomic 8-oxoG was significantly reduced in D1133L-overexpressing HEK 293T cells or PAM 3D4/21 cells (Figure 2B; Supplementary Figure S4). Moreover, overexpression of D1133L reduced the level of 8-oxoG in DNA at incremental menadione concentrations (Figure 2C). To further examine whether D1133L specifically responds to pre-existing oxidative DNA damage, HEK 293T cells were first treated with or without menadione and then transfected with Flag-D1133L plasmids. The results showed that D1133L functioned in the presence of oxidative damage (Figure 2D). These results suggested that D1133L may play a role in promoting OGG1-driven incision of 8-oxoG.

### Acetylation of D1133L is altered by oxidative stress

**Figure 3:**
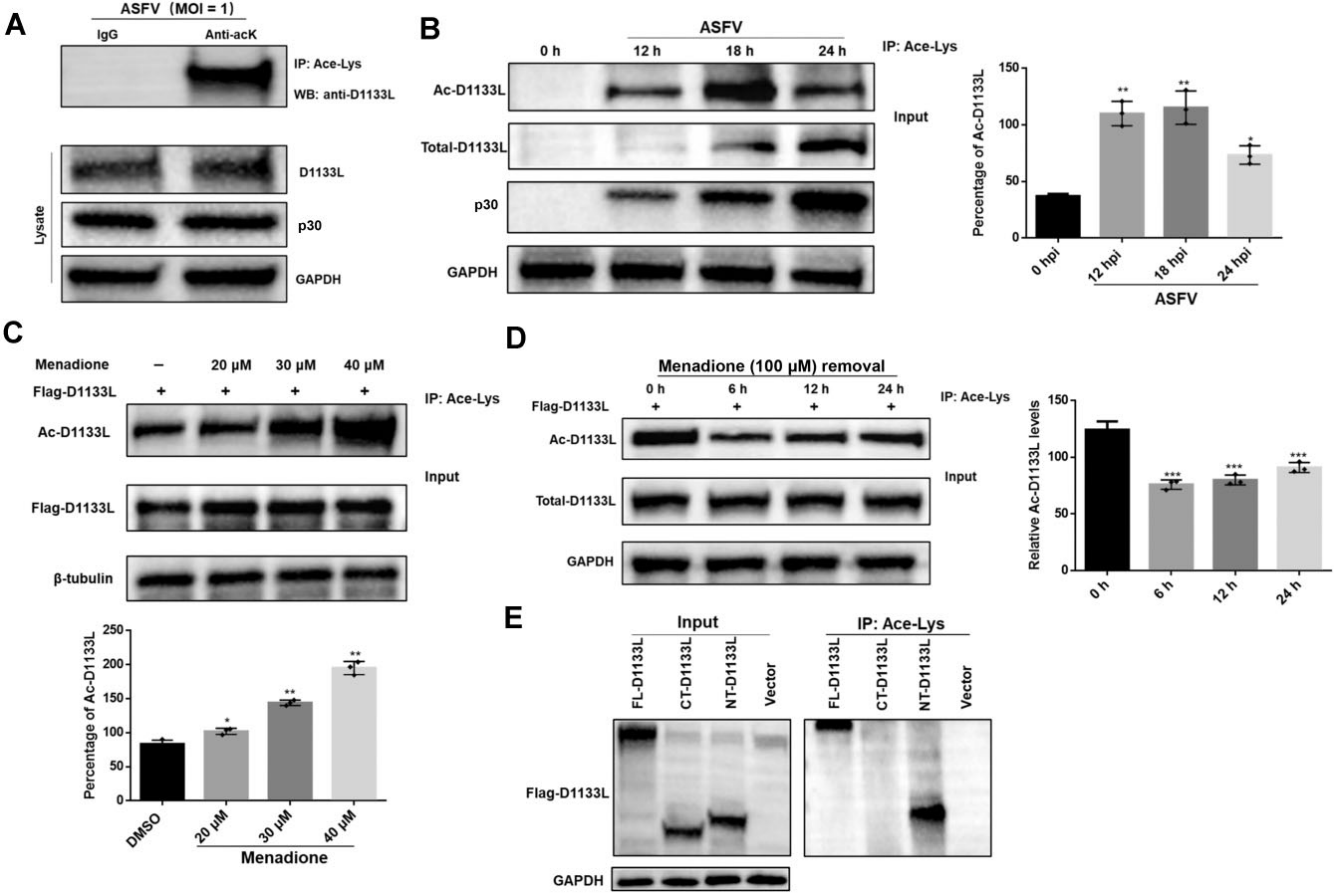
ASFV infection and menadione-induced oxidative stress alter the acetylation of D1133L. (**A**) Acetylation of endogenous D1133L in PAMs at 24 h post ASFV (MOI = 1) infection was assessed by IP using anti-acetyl-lysine antibody (Ace-Lys), followed by western blotting using anti-D1133L antibody. Input for IP was utilized to measure the levels of total D1133L, ASFV p30 protein indicating ASFV infection, and GAPDH as a loading control. (**B**) Dynamic changes of endogenous D1133L acetylation during ASFV infection. PAMs were infected with ASFV (MOI = 1) for 0, 12, 18, or 24 h. Ac-D1133L levels were normalized to total D1133L and quantified. (**C**) Concentration-dependent increase of Ac-D1133L levels in response to incremental menadione. HEK 293T cells were transfected with Flag-D1133L (2 μg) plasmids for 24 h, followed by treatment with increasing concentrations of menadione for 6 h. Whole-cell extracts were prepared for Ace-Lys IP, followed by western blotting using Flag-specific antibody, with β-tubulin as a loading control. (**D**) Ac-D1133L levels decrease after removing menadione. HEK 293T cells were transfected with Flag-D1133L (2 μg) plasmids for 24 h and treated with menadione (100 μM) for 1 h. Then, the medium was refreshed, and Ac-D1133L levels were assessed and quantified at 0, 6, 12, and 24 h. (**E**) Acetylation of NT-D1133L. HEK 293T cells were transfected with FL-D1133L, NT-D1133L, or CT-D1133L (2 μg each) for 24 h, and Ac-D1133L levels were assessed. Data are presented as mean ± SD (*n* = 3). ***P < 0.001; **P < 0.01; *P < 0.05 (*t*-test).

The acetylation of D1133L was assessed by IP of acetylated D1133L (Ac-D1133L) using anti-acetyl-lysine antibody (Ace-Lys) followed by western blotting using anti-D1133L antibody. The results confirmed that endogenous D1133L underwent acetylation modification during ASFV infection (Figure 3A). Furthermore, the acetylation level of D1133L varied with the progression of ASFV infection, significantly increasing at the early stage and slightly decreasing later (Figure 3B). Additionally, the acetylation level of exogenously introduced D1133L dose-dependently increased upon menadione exposure (Figure 3C) and gradually decreased after removal of menadione (Figure 3D). We further investigated which domain of D1133L was acetylated. The results showed that FL-D1133L and NT-D1133L could be acetylated, while CT-D1133L showed no acetylation (Figure 3E). These findings suggested that D1133L undergoes N-terminal acetylation that can be specifically triggered by oxidative stress in ASFV-infected cells.

### D1133L is a substrate for the histone acetyltransferases CBP/p300

**Figure 4:**
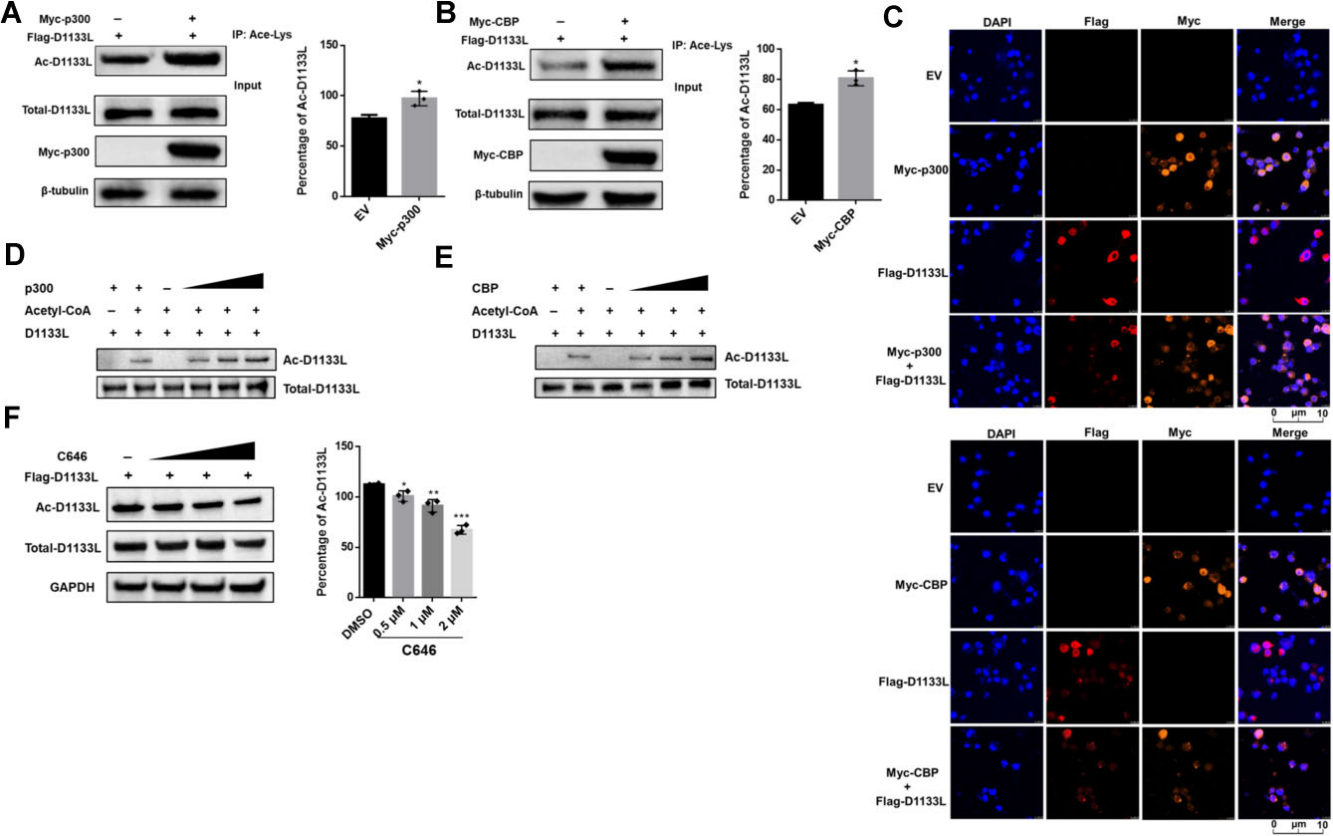
D1133L is acetylated by CBP/p300. (**A** and **B**) Acetylation of D1133L by CBP/p300 in cells. HEK 293T cells were co-transfected with Flag-D1133L (2 μg) and Myc-p300 (2 μg) or Myc-CBP (2 μg) for 24 h, and Ac-D1133L levels were assessed and quantified. (**C**) Subcellular localization of D1133L and CBP/p300. HEK 293T cells were transfected with Flag-D1133L (2 μg) and/or Myc-CBP or Myc-p300 (2 μg each) for 24 h. Confocal microscopy was employed to detect subcellular localization with anti-Flag and anti-Myc antibodies. DAPI was used to stain the nucleus. Scale bar, 10 μm. (**D** and **E**) Acetylation of D1133L by CBP/p300 *in vitro*. Recombinant His-D1133L (2.6 μg), 2 mM acetyl-CoA, and increasing amounts of recombinant p300 (0.18, 0.27, 0.36 μg) or CBP (0.2, 0.3, 0.5 μg) were incubated in acetylation buffer at 30°C for 1. Ac-D1133L levels were assessed by western blotting using anti-acetyl-lysine antibody. Total D1133L levels were determined using a His-tagged antibody. (**F**) Ac-D1133L levels decrease after treatment with the p300 inhibitor. HEK 293T cells were transfected with Flag-D1133L (2 μg) plasmids for 24 h, followed by treatment with the p300 inhibitor C646 (0, 0.5, 1, 2 μM) for 12 h, and Ac-D1133L levels were assessed and quantified. Data are presented as mean ± SD (*n* = 3). ***P < 0.001; **P < 0.01; *P < 0.05 (*t*-test).

As previously reported, helicases can be acetylated by the common acetyltransferases CBP/p300, which are highly conserved acetyltransferases and involved in DNA repair (Tini et al., 2002; Lakshmanan and Shaheer, 2020). To determine the acetyltransferase responsible for D1133L modification, we co-transfected Flag-D1133L with either Myc-CBP or Myc-p300 and monitored the acetylation level of D1133L. Both CBP and p300 increased acetylation of D1133L, confirming that CBP/p300 could acetylate D1133L (Figure 4A and B; Supplementary Figure S5). Meanwhile, we examined the subcellular localization of D1133L and CBP/p300. Previous studies indicated that ASFV replication occurs in viral cytoplasmic factories, while a portion of the ASFV genome is also present within the host cell nucleus (Simoes et al., 2015). Indeed, we observed a weak distribution of D1133L in the nucleus, partially co-localized with CBP/p300 (Figure 4C), suggesting that acetylation of D1133L may occur within the nucleus. *In vitro* acetylation assays by incubating recombinant D1133L with CBP/p300 also revealed robust acetylation of D1133L, suggesting that D1133L is a substrate for CBP/p300 (Figure 4D and E). Furthermore, we used C646 as an inhibitor of the p300 histone acetyltransferase (Chen et al., 2024). Non-toxic doses of C646 were determined in HEK 293T cells, with cell viability >70% by the concentration up to 5 μM (Supplementary Figure S3B). As expected, the level of D1133L acetylation dose-dependently decreased following C646 treatment (Figure 4F).

### D1133L is deacetylated by HDAC5

**Figure 5:**
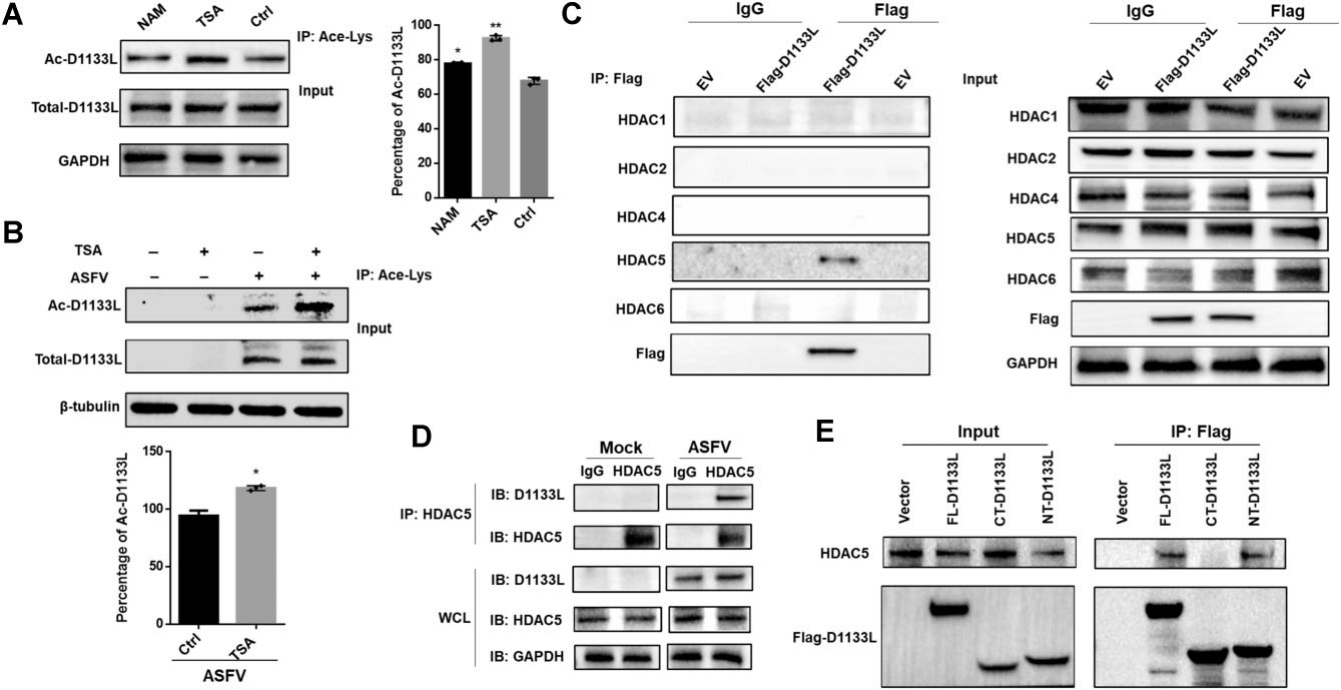
D1133L selectively interacts with HDAC5. (**A**) Changes in Ac-D1133L levels upon treatment with TSA or NAM. HEK 293T cells were transfected with Flag-D1133L (2 μg) for 24 h and then treated with the deacetylase inhibitor TSA (2 μM) or NAM (5 mM) for 6 h, and Ac-D1133L levels were assessed and quantified. (**B**) Acetylation of endogenous D1133L in PAMs treated with TSA. PAMs were treated with TSA (2 μM) and infected with ASFV (MOI = 1) for 24 h. Ac-D1133L levels were assessed and quantified. (**C**) Interaction between D1133L and HDAC5. HEK 293T cells were transfected with Flag-D1133L (2 μg) or empty vector (EV) for 24 h and subjected to Flag IP, followed by IB using anti-HDAC antibodies. (**D**) Interaction between D1133L and HDAC5 during ASFV Infection. PAMs were infected with ASFV (MOI = 1) for 24 h and subjected to HDAC5 IP, followed by IB using anti-D1133L and anti-HDAC5 antibodies. (**E**) HDAC5 interacts with NT-D1133L. HEK 293T cells were transfected with Flag-tagged FL-D1133L, NT-D1133L, or CT-D1133L (2 μg each) or empty vector for 24 h and subjected to Flag IP, followed by IB with anti-HDAC5 antibody. Data are presented as mean ± SD (*n* = 3). Statistical significance was performed using Student's *t*-test, compared with the control group. **P < 0.01; *P < 0.05.

**Figure 6:**
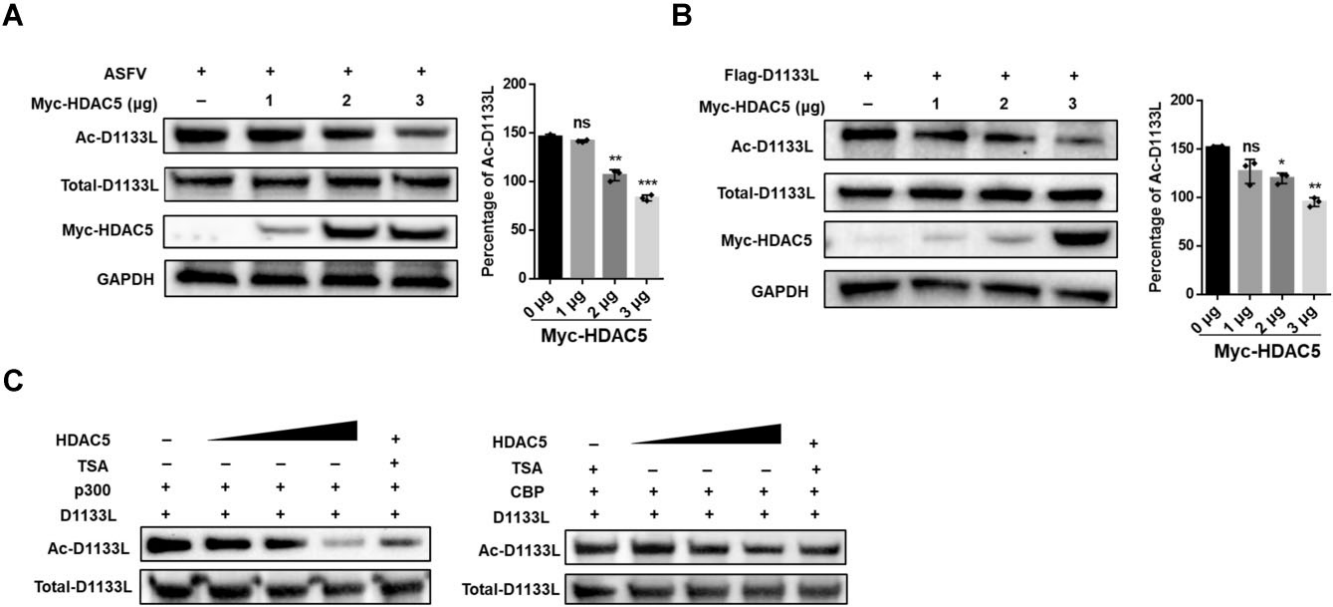
HDAC5 deacetylates D1133L. (**A**) Deacetylation of endogenous D1133L by HDAC5 in MA104 cells. MA104 cells were transfected with Myc-HDAC5 (0, 1, 2, 3 μg) for 24 h, followed by infection with ASFV (MOI = 1) for an additional 24 h, and Ac-D1133L levels were assessed and quantified. (**B**) HDAC5 deacetylates exogenous D1133L in a dose-dependent manner. HEK 293T cells were co-transfected with Flag-D1133L (2 μg) and Myc-HDAC5 (0, 1, 2, 3 μg) plasmids for 24 h, and Ac-D1133L levels were assessed and quantified. (**C**) Deacetylation of D1133L by HDAC5 *in vitro*. Recombinant His-D1133L (5.2 μg) was initially acetylated by CBP (0.5 μg) or p300 (0.36 μg) for 1 h and subsequently incubated with increasing concentrations of recombinant HDAC5 (0.25, 0.5, 1, 1 μg) or TSA (2 μM) for an additional 1 h at 30°C. Ac-D1133L levels were assessed with anti-acetyl-lysine antibody, and total D1133L levels were measured with anti-His antibody. Data are presented as mean ± SD (*n* = 3). ***P < 0.001; **P < 0.01; *P < 0.05; ns, not significant (*t*-test).

Next, we used trichostatin A (TSA), a potent and reversible inhibitor of pan-HDAC family deacetylases, and nicotinamide (NAM), an inhibitor of SIRT family deacetylases, to investigate the deacetylation of D1133L. The significantly elevated acetylation level of D1133L by TSA treatment in D1133L-overexpressing HEK 293T cells suggested the involvement of HDAC family deacetylases (Figure 5A). We also detected strongly augmented acetylation of endogenous D1133L in TSA-treated PAMs post ASFV infection (Figure 5B). Using co-IP, we detected strong interaction between HDAC5 and D1133L both exogenously and endogenously (Figure 5C and D). Furthermore, FL-D1133L and NT-D1133L, but not CT-D1133L, were co-precipitated with HDAC5 (Figure 5E; Supplementary Figure S6), manifesting that the DEAD-box domain of D1133L plays an indispensable role in its interaction and deacetylation. We then examined exogenous and endogenous D1133L acetylation after overexpressing HDAC5 and found that HDAC5 significantly decreased the acetylation level of D1133L in a dose-dependent manner (Figure 6A and B). Additionally, the deacylation assay *in vitro* reconfirmed that D1133L can be deacetylated by the recombinant HDAC5 protein (Figure 6C). Taken together, these results highlight a specific interaction between D1133L and the deacetylase HDAC5, underscoring that D1133L is a novel target of HDAC5.

### The deacetylation of D1133L by HDAC5 is associated with OGG1

**Figure 7:**
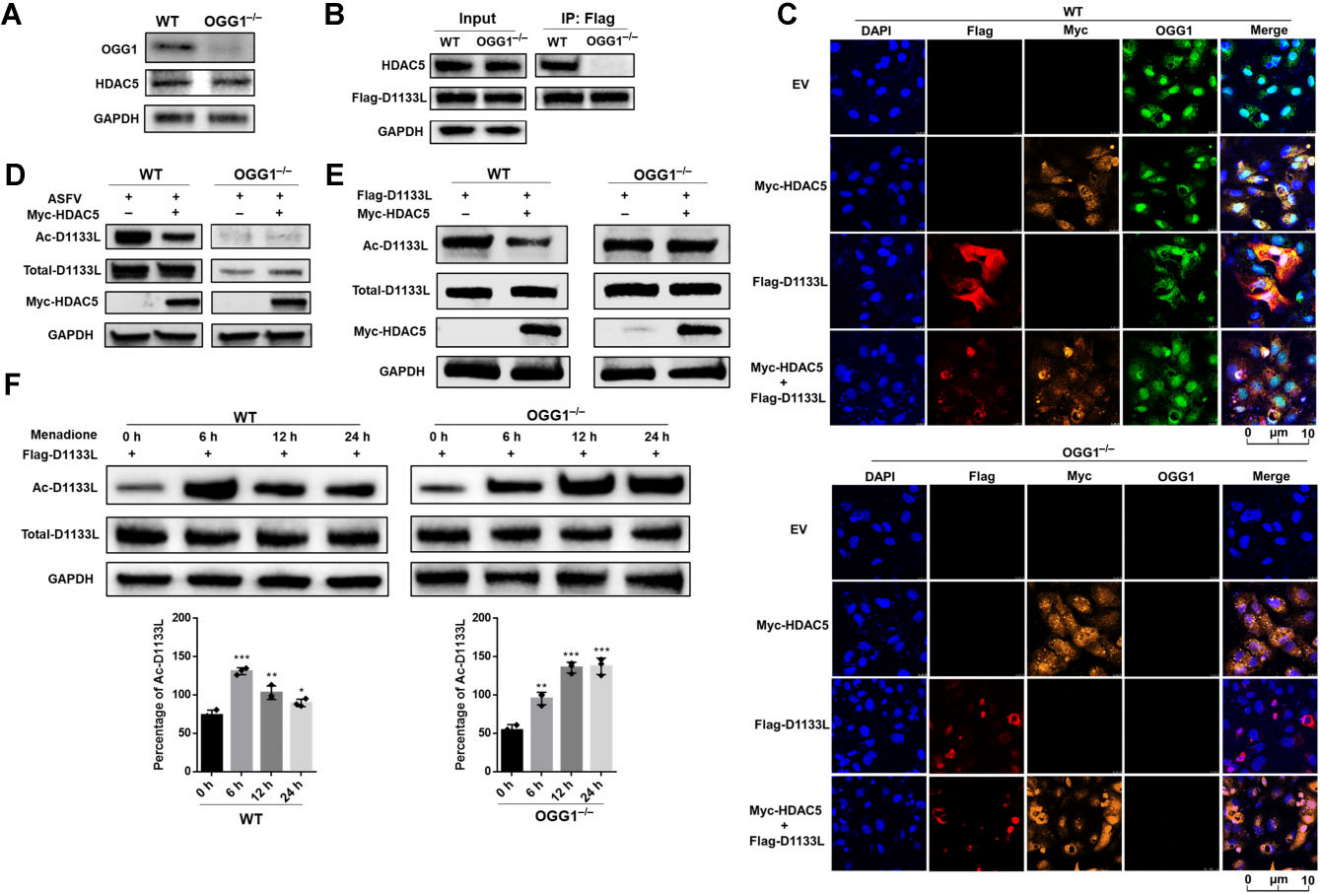
OGG1 is involved in HDAC5-mediated deacetylation of D1133L. (**A**) Western blot analysis of OGG1 and HDAC5 levels in WT and OGG1^–/–^ MA104 cells. (**B**) Interaction between D1133L and HDAC5 only in WT MA104 cells. Lysates of WT and OGG1^–/–^ MA104 cells were transfected with Flag-D1133L (2 μg) plasmids for 24 h and subjected to Flag IP, followed by IB with anti-HDAC5 antibody. (**C**) Co-localization of D1133L, OGG1, and HDAC5. WT and OGG1^–/–^ MA104 cells were transfected with Flag-D1133L (2 μg) and/or Myc-HDAC5 (2 μg) for 24 h. Confocal microscopy was used to detect the co-localization by using anti-Flag, anti-OGG1, and 647-conjugated Myc tag monoclonal antibodies. DAPI was used to stain the nucleus. Scale bar, 10 μm. (**D**) Deacetylation of endogenous D1133L by HDAC5 in cells. WT and OGG1^–/–^ MA104 cells were transfected with Myc-HDAC5 (2 μg) for 24 h, followed by ASFV infection (MOI = 1) for an additional 24 h, and Ac-D1133L levels were assessed. (**E**) Deacetylation of transgenically expressed D1133L by HDAC5. WT and OGG1^–/–^ MA104 cells were transfected with Flag-D1133L (2 μg) and/or Myc-HDAC5 (2 μg) plasmids, and Ac-D1133L levels were assessed. (**F**) Deacetylation of D1133L in cells following removal of menadione treatment. WT and OGG1^–/–^ MA104 cells were transfected with Flag-D1133L (2 μg) plasmids for 24 h and treated with menadione (50 μM) for 2 h. Then, the medium was refreshed, and Ac-D1133L levels were assessed and quantified at the indicated time points. Data are presented as mean ± SD (*n* = 3). ***P < 0.001; **P < 0.01; *P < 0.05 (*t*-test).

To understand the role of OGG1 in deacetylation of D1133L by HDAC5, we used CRISPR–Cas9 to knock out OGG1 in MA104 cells. HDAC5 protein levels in wild-type (WT) and OGG1 knockout (OGG1^–/–^) cells were comparable, indicating that knockout of OGG1 did not influence HDAC5 level (Figure 7A; Supplementary Figure S7A). However, the interaction between D1133L and HDAC5 was blocked in OGG1^–/–^ cells (Figure 7B). Confocal microscopy was performed to examine the subcellular localization of D1133L and HDAC5 in WT and OGG1^–/–^ cells. We observed that OGG1, HDAC5, and D1133L were primarily co-localized in the cytoplasm, but the absence of OGG1 attenuated the co-localization of HDAC5 with D1133L (Figure 7C), suggesting that OGG1 might impact the deacetylation of D1133L.

In support of this possibility, the protein level of endogenous D1133L and its acetylation were scarcely detectable in OGG1^–/–^ MA104 cells (Figure 7D). Consistent with previously study demonstrating that OGG1 knockdown suppresses ASFV replication (Fan et al., 2023), the deletion of OGG1 nearly halted the replication of ASFV (Supplementary Figure S7B).

The acetylation of exogenous D1133L was decreased by overexpressing HDAC5 in WT cells, but no change was observed in OGG1^–/–^ cells, indicating that HDAC5-mediated D1133L deacetylation was abolished by OGG1 knockout (Figure 7E). Additionally, Ac-D1133L levels increased after menadione removal (6 h recovery) in both WT and OGG1^–/–^ cells, but the following decreases (indicating deacetylation) did not occur in OGG1^–/–^ cells, confirming that HDAC5-mediated deacetylation of D1133L is counteracted by the knockout of OGG1 (Figure 7F). Previous studies suggested that HDAC5-mediated deacetylation regulates the PARP1-dependent DNA damage response and promotes the efficient recruitment of DNA repair factors to the damaged sites (Tyagi and Das, 2024). Based on our results, OGG1 is involved in the deacetylation of D1133L by HDAC5, which may play a pivotal role in OGG1-mediated BER.

### D1133L promotes the incision of intrahelical 8-oxoG by OGG1

**Figure 8:**
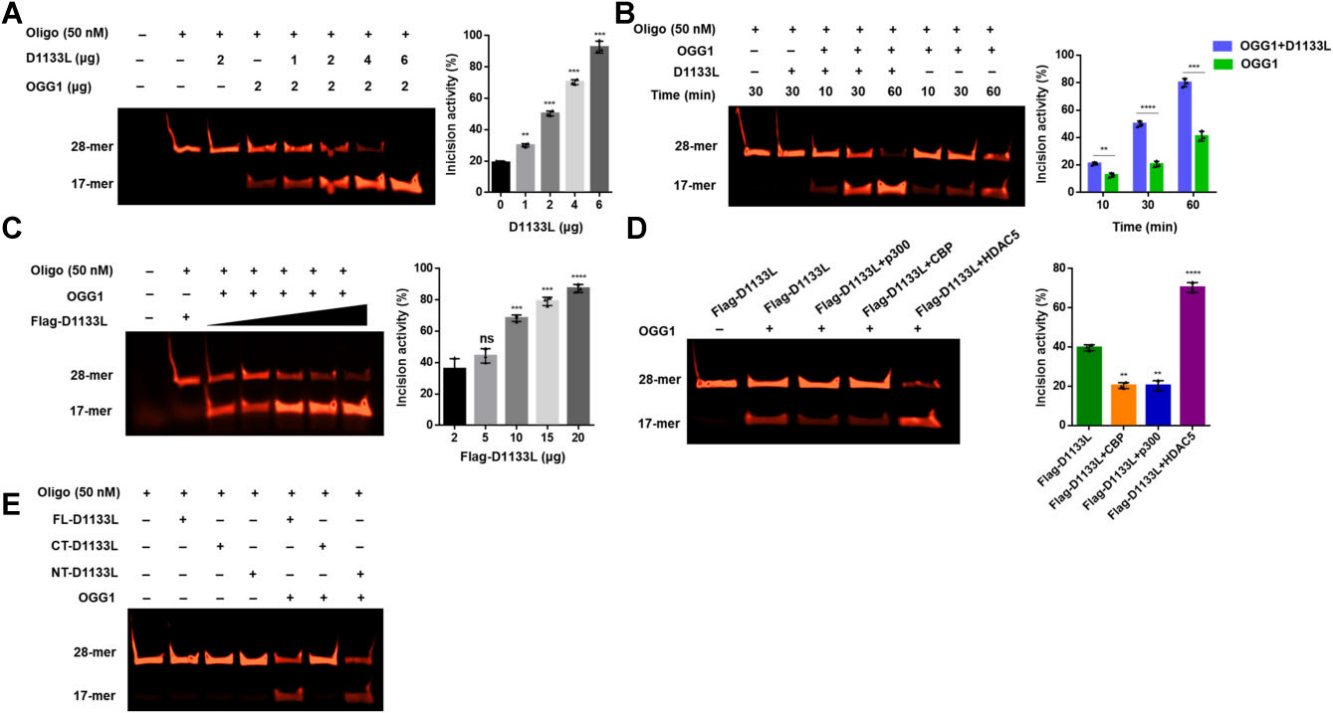
D1133L enhances the incision activity of OGG1 on 8-oxoG. (**A**) D1133L promotes 8-oxoG incision by OGG1 in a dose-dependent manner. Cy5-labeled oligonucleotides (50 nM) were incubated with increasing concentrations of recombinant D1133L and/or OGG1 for 30 min at 37°C in the reaction buffer. The 8-oxoG incision was analyzed by gel electrophoresis, and the incision activity of OGG1 was quantified. (**B**) D1133L promotes 8-oxoG incision by OGG1 in a time-dependent manner. Recombinant D1133L (2 μg) and/or OGG1 (2 μg) were incubated with 50 nM Cy5-labeled oligonucleotides for the indicated time at 37°C and analyzed by gel electrophoresis. The incision activity of OGG1 was quantified. (**C**) Immunopurified Flag-D1133L promotes 8-oxoG incision by OGG1. HEK 293T cells were transfected with Flag-D1133L (2 μg) for 24 h. Whole-cell extracts were incubated with anti-Flag beads for 6 h at 4°C. Then, Flag-D1133L was eluted by IP buffer supplemented with 1.5 μg/μl Flag-peptide. Increasing concentrations of immunopurified Flag-D1133L and OGG1 (2 μg) were incubated with oligonucleotides for 1 h at 37°C. (**D**) Incision activity of OGG1 is induced by deacetylation of D1133L. HEK 293T cells were co-transfected with Flag-D1133L (2 μg) and/or p300, CBP, or HDAC5 (2 μg each) for 24 h. The immunopurified Flag-D1133L proteins were collected and used for the 8-oxoG incision assay. (**E**) NT-D1133L facilitates OGG1-driven 8-oxoG incision. HEK 293T cells were transfected with FL-D1133L (2 μg), NT-D1133L (2 μg), CT-D1133L (2 μg), or empty vector for 24 h. Flag-tagged proteins were collected, and FL-D1133L (5 μg), NT-D1133L (5 μg), or CT-D1133L (2 μg) was incubated with oligonucleotides and/or OGG1 (2 μg) for 1 h at 37°C. The 8-oxoG incision was analyzed by gel electrophoresis. Data are shown as mean ± SD (*n* = 3). ****P < 0.0001; ***P < 0.001; **P < 0.01; *P < 0.05; ns, not significant (*t*-test).

To gain insight into the biological function of D1133L, we incubated recombinant D1133L and OGG1 with Cy5-labeled 8-oxoG-containing oligonucleotides (probe) and monitored the incision of labeled DNA by gel electrophoresis. The results showed that D1133L promoted 8-oxoG incision by OGG1 in a concentration- and time-dependent manner *in vitro* (Figure 8A and B). Furthermore, the incision activity of OGG1 on 8-oxoG-containing probe was enhanced upon increasing amounts of immunopurified Flag-D1133L from cells (Figure 8C).

To determine whether the incision activity was regulated by the acetylation status of D1133L, we examine the incision activity of OGG1 in the presence of Flag-D1133L purified from control cells or cells overexpressing CBP, p300, or HDAC5. The hypoacetylated Flag-D1133L from HDAC5-overexpressing cells significantly enhanced the incision activity of OGG1, while the hyperacetylated Flag-D1133L from CBP/p300-overexpressing cells reduced the incision activity (Figure 8D; Supplementary Figure S8). Moreover, the N-terminal region of D1133L was necessary for its ability to promote 8-oxoG incision by OGG1 (Figure 8E). These results demonstrate that acetylation compromises D1133L's ability to stimulate OGG1-driven 8-oxoG incision, while deacetylation of D1133L augments such ability.

## Discussion

DEAD-box RNA helicases have been confirmed to play important roles in various viral infections, serving either as mediators of anti-viral innate immunity or as essential components for viral replication (Steimer and Klostermeier, 2012; Feng et al., 2017). Previous studies reported that certain RNA helicases also show unique DNA helicase activity, implicating their involvement in RNA and DNA metabolism (Fuller-Pace, 2013; Russell, 2015). DEAD-box RNA helicases are known to participate in DNA damage repair processes such as DSB repair and ribonucleotide excision repair, contributing to the maintenance of genome stability (Riva et al., 2020; Chakraborty and Hiom, 2021; Bader et al., 2022). It has been demonstrated that stress induces the expression of DEAD-box helicases, which play a crucial role in modulating antioxidant defense machinery (Tuteja et al., 2013, 2014).

In this study, we document a unique perspective on the involvement of ASFV DEAD-box RNA helicase D1133L in BER. We confirmed that ASFV D1133L, in particular its N-terminal DEAD-box region, selectively interacts with OGG1, thereby participating in the elimination of genomic 8-oxoG. Our previous study demonstrated that ASFV infection induces the production of ROS and 8-oxoG, leading to an increased OGG1 level for the repair of DNA damage (Fan et al., 2023). Indeed, our observations indicate that D1133L overexpression decreases the levels of 8-oxoG under oxidative stress and in ASFV-infected cells (Figure 2). Consequently, we emphasize, for the first time, the role of helicase D1133L in maintaining genome stability by mitigating the accumulation of 8-oxoG. According to the current models, the activity of RNA helicase is intricately associated with acetylation and deacetylation processes. For example, the acetylation of DDX21 by CBP inhibits its activity, while deacetylation by SIRT7 enhances the helicase activity (Song et al., 2017). In the case of RECQL4, acetylation and its binding ability to OGG1 are enhanced under oxidative stress, a process counteracted by SIRT1 (Duan et al., 2020). Intriguingly, our discovery reveals that D1133L is an acetylated protein susceptible to acetylation by CBP/p300, both of which are involved in chromatin remodeling and DNA repair via intrinsic histone acetyltransferase activity (Tini et al., 2002).

Intracellular protein modification represents a dynamic balance that governs various physiological and pathological processes, with acetyltransferases and deacetylases playing crucial roles in maintaining this equilibrium (Peserico and Simone, 2011; Wan et al., 2020). We identified a specific interaction between D1133L and HDAC5, which is a potential host factor supporting viral biosynthesis (Taha et al., 2020). Post-translational modifications contribute to dynamically controlling protein function (Bennetzen et al., 2013), e.g. acetylation leads to a transfer of protein from the nucleus to the cytoplasm (Dietschy et al., 2009). Remarkably, D1133L is co-localized with CBP/p300 in the nucleus, while most of D1133L interacts with OGG1 and HDAC5 in the cytoplasm. Based on these findings, we speculate that acetylation of D1133L occurs in the nucleus, while deacetylation primarily takes place in the cytoplasm. Additionally, acetylation may be associated with its export to the cytoplasm, which warrants further investigation. In line with the involvement of NT-D1133L in OGG1 binding, we observed HDAC5 binding to both FL-D1133L and NT-D1133L, suggesting that the deacetylation of D1133L by HDAC5 may be linked to the activity of DEAD-box RNA helicase. Previous research already revealed the role of HDAC5 in the suppression of oxidative stress (Hu et al., 2019). Our findings indicate that OGG1 is required for the deacetylation of D1133L by HDAC5.

OGG1 recognizes the oxidatively modified heterocyclic bases in DNA and initiates the first step of repairing 8-oxoG by cleaving the N-glycosylic bond, releasing the damaged base and forming an apurinic/apyrimidinic site (Michel et al., 2022). The activity of OGG1 in 8-oxoG repair can be regulated by p300 (Bhakat et al., 2006). Additionally, SIRT3-mediated deacetylation of OGG1 controls its incision activity (Cheng et al., 2013). HDAC1 also plays important roles in OGG1-mediated 8-oxoG repair (Pao et al., 2020). Our results support the notion that D1133L modulates 8-oxoG incision by interacting with OGG1, and HDAC5 binding to D1133L regulates this process by altering its acetylation status. However, further research is needed to clarify whether and how OGG1 recruits HDAC5 to deacetylate D1133L.

Virus–host protein interaction is a crucial mechanism through which viruses exploit host proteins to manipulate cellular pathways and facilitate their replication (Shah et al., 2022). Commonly, viruses seek to hijack host factors to support infection, while host cells attempt to impede or slow down the viral synthetic machinery (Khadka et al., 2011). Recent study has revealed that 8-oxoG is not merely a byproduct of ROS but serves as a strategic adaptation of the respiratory syncytial virus genome to maintain genetic fidelity by hijacking OGG1 (Pan et al., 2024). As a part of an anti-viral strategy, hyperacetylation of D1133L by host acetyltransferases inhibits its activity, while ASFV hijacks the deacetylase HDAC5 through binding to OGG1 to keep D1133L in a hypoacetylated state. Therefore, we propose that ASFV employs a strategy of hijacking OGG1 and HDAC5 through D1133L to regulate DNA BER pathway for the maintenance of viral DNA fidelity and efficient virus propagation. However, as a viral protein, the function of D1133L is multifaceted. D1133L may assist OGG1 in the removal of 8-oxoG from host nuclear DNA and mitochondrial DNA or possibly from its own viral genome. The ultimate purpose behind these phenomena is to promote viral replication.

Collectively, the present study revealed a novel function of D1133L in DNA damage repair, highlighting the functional connection between D1133L and OGG1 (Supplementary Figure S9). This discovery established a novel post-transcriptional regulatory mechanism that governs the activity of D1133L in BER and safeguards genome integrity to maintain the normal expression of virus and host genes, which are key to ASFV replication.

## Materials and methods

### Biosafety and ethical statement

All experiments involving ASFV were conducted at the Biosafety Level 3 (BSL-3) laboratory of Lanzhou Veterinary Research Institute (LVRI), Chinese Academy of Agricultural Sciences (CAAS). The handling of the animals in this study adhered to the animal ethics procedures and guidelines of LVRI, CAAS (approval number: LVRIAEC-2023-043). This laboratory is accredited by China National Accreditation Service for Conformity Assessment (CNAS) and approved by the Ministry of Agriculture and Rural Affairs. To mitigate any potential risks in the laboratory, strict adherence to protocols is enforced, with all activities monitored by professional staff at LVRI. Additionally, local and central government authorities conduct random inspections without prior notification.

### Cells and viruses

HEK 293T cells were cultured in Dulbecco's modified Eagle medium (DMEM; 11965092, Gibco) supplemented with 10% fetal bovine serum (FBS; 10091148, Gibco) and 100 IU penicillin–streptomycin (15070063, Invitrogen) at 37°C in a 5% CO_2_ atmosphere. OGG1^–/–^ MA104 cell lines were constructed locally by CRISPR–Cas9 technology. WT and OGG1^–/–^ MA104 cells were cultured in RPMI 1640 medium (0013219, BI) supplemented with 5 μg/ml puromycin (540411, Sigma) at 37°C in a humidified 5% CO_2_ incubator. The immortalized PAM line 3D4/21 (CRL-2843) and primary PAMs from 30-day-old healthy pigs were cultured in RPMI 1640 medium (11875085, Gibco) supplemented with 10% FBS and 1% penicillin‒streptomycin. ASFV CN/SC/2019 was isolated, identified, and maintained in the BSL-3 laboratory of the African Swine Fever Regional Laboratory of China (Lanzhou), LVRI, CAAS.

### Reagents and antibodies

Cy5-labeled oligonucleotides were synthesized by Sangon Biotech, China. TSA (V900931), NAM (N0636), menadione sodium bisulfite (M2518), C646 (328968-36-1), and acetyl-CoA (A2056) were purchased from Sigma-Aldrich. Recombinant D1133L was constructed by GenScript Inc. OGG1-6×His fusion protein (Ag7320) and HDAC5-GST fusion protein (Ag29122) were purchased from Proteintech Group, China. CREB binding protein (BML-SE425) and p300 protein (BML-SE451) were purchased from Enzo Life Science.

The antibodies used in this study were as follows: anti-OGG1 (PA1-31402, Thermo Fisher Scientific), anti-acetyl-lysine (9441, Cell Signaling Technology), anti-HDAC1 (PA1-860, Invitrogen), anti-HDAC2 (PA1-861, Invitrogen), and anti-HDAC4 (5392S, Cell Signaling Technology). Anti-HDAC5 (16166-1-AP), anti-HDAC6 (12834-1-AP), anti-NTHL-1 (11154-1-AP), anti-Myc (60003-2-Ig), anti-Flag (66008-4-Ig, 20543-1-AP), anti-HA (66006-2-Ig), anti-GAPDH (10494-1-AP), anti-β-tubulin (10094-1-AP), horseradish peroxidase (HRP)-conjugated goat anti-rabbit secondary antibody (SA00001-2), HRP-conjugated goat anti-mouse secondary antibody (SA00013-3), and 647-conjugated Myc tag monoclonal antibody were obtained from Proteintech Group. Goat anti-rabbit IgG (H+L) (Alexa Fluor 488, A32731) and goat anti-mouse IgG (H+L) (Alexa Fluor 555, A28180) secondary antibodies were purchased from Invitrogen. Anti-D1133L, anti-p30, and anti-p72 rabbit polyclonal antibodies were provided by the African Swine Fever Regional Laboratory of China (Lanzhou), LVRI, CAAS.

### Statistical analysis

All data are presented as mean ± standard deviation (SD). The statistical significance of the difference between groups was analyzed using Prism 8.0 software (GraphPad). The criterion *P*-value < 0.05 was considered statistically significant (*P < 0.05, **P < 0.01, ***P < 0.001, ****P < 0.0001).

More details for routine methods can be found in Supplementary material.

## Supplementary Material

mjaf029_Supplemental_File

## Notes

**Author contributions:** conceptualization: Q.N. and J.F. Data curation: J.F., X.Z., and S.G. Formal analysis: J.Y. Funding acquisition: Q.N., H.Y., and G.G. Investigation: Z.T. Supervision: Q.N., I.B., H.Y., J.L., and Q.Z. Writing—original draft: J.F. and Q.N. Writing—review and editing: I.B. and H.Y.

## References

[bib1] Abbasi S., Schild-Poulter C. (2019). Mapping the Ku interactome using proximity-dependent biotin identification in human cells. J. Proteome Res. 18, 1064–1077. 30585729 10.1021/acs.jproteome.8b00771

[bib2] Bader A.S., Luessing J., Hawley B.R. et al. (2022). DDX17 is required for efficient DSB repair at DNA:RNA hybrid deficient loci. Nucleic Acids Res. 50, 10487–10502. 36200807 10.1093/nar/gkac843 PMC9561282

[bib3] Balakrishnan L., Stewart J., Polaczek P. et al. (2010). Acetylation of Dna2 endonuclease/helicase and flap endonuclease 1 by p300 promotes DNA stability by creating long flap intermediates. J. Biol. Chem. 285, 4398–4404. 20019387 10.1074/jbc.M109.086397 PMC2836044

[bib4] Bennetzen M.V., Larsen D.H., Dinant C. et al. (2013). Acetylation dynamics of human nuclear proteins during the ionizing radiation-induced DNA damage response. Cell Cycle 12, 1688–1695. 23656789 10.4161/cc.24758 PMC3713127

[bib5] Bhakat K.K., Mokkapati S.K., Boldogh I. et al. (2006). Acetylation of human 8-oxoguanine-DNA glycosylase by p300 and its role in 8-oxoguanine repair *in vivo*. Mol. Cell. Biol. 26, 1654–1665. 16478987 10.1128/MCB.26.5.1654-1665.2006 PMC1430230

[bib6] Cargill M., Venkataraman R., Lee S. (2021a). DEAD-box RNA helicases and genome stability. Genes 12, 1471. 34680866 10.3390/genes12101471 PMC8535883

[bib7] Cargill M.J., Morales A., Ravishankar S. et al. (2021). RNA helicase, DDX3X, is actively recruited to sites of DNA damage in live cells. DNA Repair 103, 103137. 34083132 10.1016/j.dnarep.2021.103137 PMC8544569

[bib8] Chakraborty P., Hiom K. (2021). DHX9-dependent recruitment of BRCA1 to RNA promotes DNA end resection in homologous recombination. Nat. Commun. 12, 4126. 34226554 10.1038/s41467-021-24341-z PMC8257769

[bib9] Chan Y.A., Hieter P., Stirling P.C. (2014). Mechanisms of genome instability induced by RNA-processing defects. Trends Genet. 30, 245–253. 24794811 10.1016/j.tig.2014.03.005 PMC4039741

[bib10] Chen Y.F., Rahman A., Sax J.L. et al. (2024). C646 degrades Exportin-1 to modulate p300 chromatin occupancy and function. Cell Chem. Biol. 31, 1363–1372.e8. 38917791 10.1016/j.chembiol.2024.05.016 PMC11268802

[bib11] Cheng Y., Ren X., Gowda A.S. et al. (2013). Interaction of Sirt3 with OGG1 contributes to repair of mitochondrial DNA and protects from apoptotic cell death under oxidative stress. Cell Death. Dis. 4, e731. 23868064 10.1038/cddis.2013.254 PMC3730425

[bib12] Dietschy T., Shevelev I., Pena-Diaz J. et al. (2009). P300-mediated acetylation of the Rothmund–Thomson-syndrome gene product RECQL4 regulates its subcellular localization. J. Cell Sci. 122, 1258–1267. 19299466 10.1242/jcs.037747

[bib13] Duan S., Han X., Akbari M. et al. (2020). Interaction between RECQL4 and OGG1 promotes repair of oxidative base lesion 8-oxoG and is regulated by SIRT1 deacetylase. Nucleic Acids Res. 48, 6530–6546. 32432680 10.1093/nar/gkaa392 PMC7337523

[bib14] Dutertre M., Lambert S., Carreira A. et al. (2014). DNA damage: RNA-binding proteins protect from near and far. Trends Biochem. Sci. 39, 141–149. 24534650 10.1016/j.tibs.2014.01.003

[bib15] Fan J., Lv X., Yang S. et al. (2023). OGG1 inhibition suppresses African swine fever virus replication. Virol. Sin. 38, 96–107. 36435451 10.1016/j.virs.2022.11.006 PMC10006199

[bib16] Feng T., Sun T., Li G. et al. (2017). DEAD-box helicase DDX25 is a negative regulator of type I interferon pathway and facilitates RNA virus infection. Front. Cell. Infect. Microbiol. 7, 356. 28824886 10.3389/fcimb.2017.00356 PMC5543031

[bib17] Fouquerel E., Barnes R.P., Uttam S. et al. (2019). Targeted and persistent 8-oxoguanine base damage at telomeres promotes telomere loss and crisis. Mol. Cell 75, 117–130.e6. 31101499 10.1016/j.molcel.2019.04.024 PMC6625854

[bib18] Freitas F.B., Frouco G., Martins C. et al. (2019). The QP509L and Q706L superfamily II RNA helicases of African swine fever virus are required for viral replication, having non-redundant activities. Emerg. Microbes Infect. 8, 291–302. 30866783 10.1080/22221751.2019.1578624 PMC6455146

[bib19] Fuller-Pace F.V. (2013). DEAD box RNA helicase functions in cancer. RNA Biol. 10, 121–132. 23353573 10.4161/rna.23312 PMC3590229

[bib20] Gaudreault N.N., Madden D.W., Wilson W.C. et al. (2020). African swine fever virus: an emerging DNA arbovirus. Front. Vet. Sci. 7, 215. 32478103 10.3389/fvets.2020.00215 PMC7237725

[bib21] Hao Y., Yang J., Yang B. et al. (2022). Identification and analysis of the interaction network of African swine fever virus D1133L with host proteins. Front. Microbiol. 13, 1037346. 36406406 10.3389/fmicb.2022.1037346 PMC9673173

[bib22] Hodroj D., Recolin B., Serhal K. et al. (2017). An ATR-dependent function for the Ddx19 RNA helicase in nuclear R-loop metabolism. EMBO J. 36, 1182–1198. 28314779 10.15252/embj.201695131 PMC5412905

[bib23] Hu T., Schreiter F.C., Bagchi R.A. et al. (2019). HDAC5 catalytic activity suppresses cardiomyocyte oxidative stress and NRF2 target gene expression. J. Biol. Chem. 294, 8640–8652. 30962285 10.1074/jbc.RA118.007006 PMC6544848

[bib24] Jun Y.W., Albarran E., Wilson D.L. et al. (2022). Fluorescence imaging of mitochondrial DNA base excision repair reveals dynamics of oxidative stress responses. Angew. Chem. Int. Ed. Engl. 61, e202111829. 34851014 10.1002/anie.202111829 PMC8792287

[bib25] Khadka S., Vangeloff A.D., Zhang C. et al. (2011). A physical interaction network of dengue virus and human proteins. Mol. Cell. Proteomics 10, M111–M12187. 10.1074/mcp.M111.012187 PMC3237087 21911577

[bib26] Lakshmanan M.D., Shaheer K. (2020). Endocrine disrupting chemicals may deregulate DNA repair through estrogen receptor mediated seizing of CBP/p300 acetylase. J. Endocrinol. Invest. 43, 1189–1196. 32253726 10.1007/s40618-020-01241-5

[bib27] Li L., Monckton E.A., Godbout R. (2008). A role for DEAD box 1 at DNA double-strand breaks. Mol. Cell. Biol. 28, 6413–6425. 18710941 10.1128/MCB.01053-08 PMC2577411

[bib28] Michel M., Benitez-Buelga C., Calvo P.A. et al. (2022). Small-molecule activation of OGG1 increases oxidative DNA damage repair by gaining a new function. Science 376, 1471–1476. 35737787 10.1126/science.abf8980

[bib29] Mooney S.M., Goel A., D'Assoro A.B. et al. (2010). Pleiotropic effects of p300-mediated acetylation on p68 and p72 RNA helicase. J. Biol. Chem. 285, 30443–30452. 20663877 10.1074/jbc.M110.143792 PMC2945537

[bib30] Osipov A., Chigasova A., Yashkina E. et al. (2023). Residual foci of DNA damage response proteins in relation to cellular senescence and autophagy in X-ray irradiated fibroblasts. Cells 12, 1209. 37190118 10.3390/cells12081209 PMC10136818

[bib31] Owttrim G.W. (2006). RNA helicases and abiotic stress. Nucleic Acids Res. 34, 3220–3230. 16790567 10.1093/nar/gkl408 PMC1484253

[bib32] Pan L., Wang K., Hao W. et al. (2024). 8-Oxoguanine DNA Glycosylase1 conceals oxidized guanine in nucleoprotein-associated RNA of respiratory syncytial virus. PLoS Pathog. 20, e1012616. 39413143 10.1371/journal.ppat.1012616 PMC11515973

[bib33] Pao P.C., Patnaik D., Watson L.A. et al. (2020). HDAC1 modulates OGG1-initiated oxidative DNA damage repair in the aging brain and Alzheimer's disease. Nat. Commun. 11, 2484. 32424276 10.1038/s41467-020-16361-y PMC7235043

[bib34] Peserico A., Simone C. (2011). Physical and functional HAT/HDAC interplay regulates protein acetylation balance. J. Biomed. Biotechnol. 2011, 371832. 21151613 10.1155/2011/371832 PMC2997516

[bib35] Polo S.E., Jackson S.P. (2011). Dynamics of DNA damage response proteins at DNA breaks: a focus on protein modifications. Genes Dev. 25, 409–433. 21363960 10.1101/gad.2021311 PMC3049283

[bib36] Ramirez-Medina E., Vuono E.A., Pruitt S. et al. (2021). Evaluation of an ASFV RNA helicase gene a859L for virus replication and swine virulence. Viruses 14, 10. 35062213 10.3390/v14010010 PMC8777736

[bib37] Riva V., Garbelli A., Casiraghi F. et al. (2020). Novel alternative ribonucleotide excision repair pathways in human cells by DDX3X and specialized DNA polymerases. Nucleic Acids Res. 48, 11551–11565. 33137198 10.1093/nar/gkaa948 PMC7672437

[bib38] Russell R. (2015). Unwinding the mechanisms of a DEAD-box RNA helicase in cancer. J. Mol. Biol. 427, 1797–1800. 25836982 10.1016/j.jmb.2015.03.009 PMC4740914

[bib39] Saito M., Iestamantavicius V., Hess D. et al. (2021). Monitoring acetylation of the RNA helicase DDX3X, a protein critical for formation of stress granules. Methods Mol. Biol. 2209, 217–234. 33201472 10.1007/978-1-0716-0935-4_14

[bib40] Shah P.S., Beesabathuni N.S., Fishburn A.T. et al. (2022). Systems biology of virus–host protein interactions: from hypothesis generation to mechanisms of replication and pathogenesis. Annu. Rev. Virol. 9, 397–415. 35576593 10.1146/annurev-virology-100520-011851 PMC10150767

[bib41] Shao Z., Su S., Yang J. et al. (2023). Structures and implications of the C962R protein of African swine fever virus. Nucleic Acids Res. 51, 9475–9490. 37587714 10.1093/nar/gkad677 PMC10516667

[bib42] Simoes M., Martins C., Ferreira F. (2015). Early intranuclear replication of African swine fever virus genome modifies the landscape of the host cell nucleus. Virus Res. 210, 1–7. 26183880 10.1016/j.virusres.2015.07.006

[bib43] Song C., Hotz-Wagenblatt A., Voit R. et al. (2017). SIRT7 and the DEAD-box helicase DDX21 cooperate to resolve genomic R loops and safeguard genome stability. Genes Dev. 31, 1370–1381. 28790157 10.1101/gad.300624.117 PMC5580657

[bib44] Steimer L., Klostermeier D. (2012). RNA helicases in infection and disease. RNA Biol. 9, 751–771. 22699555 10.4161/rna.20090

[bib45] Szeliga M., Rola R. (2022). Menadione potentiates auranofin-induced glioblastoma cell death. Int. J. Mol. Sci. 23, 15712. 36555352 10.3390/ijms232415712 PMC9778806

[bib46] Tabassum S., Ghosh M.K. (2023). DEAD-box RNA helicases with special reference to p68: unwinding their biology, versatility, and therapeutic opportunity in cancer. Genes Dis. 10, 1220–1241. 37397539 10.1016/j.gendis.2022.02.008 PMC10310985

[bib47] Taha T.Y., Anirudhan V., Limothai U. et al. (2020). Modulation of hepatitis B virus pregenomic RNA stability and splicing by histone deacetylase 5 enhances viral biosynthesis. PLoS Pathog. 16, e1008802. 32822428 10.1371/journal.ppat.1008802 PMC7467325

[bib48] Tanner N.K., Linder P. (2001). DExD/H box RNA helicases: from generic motors to specific dissociation functions. Mol. Cell 8, 251–262. 11545728 10.1016/s1097-2765(01)00329-x

[bib49] Tini M., Benecke A., Um S.J. et al. (2002). Association of CBP/p300 acetylase and thymine DNA glycosylase links DNA repair and transcription. Mol. Cell 9, 265–277. 11864601 10.1016/s1097-2765(02)00453-7

[bib50] Tuteja N., Banu M.S., Huda K.M. et al. (2014). Pea p68, a DEAD-box helicase, provides salinity stress tolerance in transgenic tobacco by reducing oxidative stress and improving photosynthesis machinery. PLoS One 9, e98287. 24879307 10.1371/journal.pone.0098287 PMC4039504

[bib51] Tuteja N., Sahoo R.K., Garg B. et al. (2013). OsSUV3 dual helicase functions in salinity stress tolerance by maintaining photosynthesis and antioxidant machinery in rice (Oryza sativa L. Cv. IR64). Plant J. 76, 115–127. 23808500 10.1111/tpj.12277

[bib52] Tyagi W., Das S. (2024). Temporal regulation of acetylation status determines PARP1 role in DNA damage response and metabolic homeostasis. Sci. Adv. 10, 7720. 10.1126/sciadv.ado7720 PMC11488539 39423262

[bib53] Wan X., Wang C., Huang Z. et al. (2020). Cisplatin inhibits SIRT3-deacetylation MTHFD2 to disturb cellular redox balance in colorectal cancer cell. Cell Death. Dis. 11, 649. 32811824 10.1038/s41419-020-02825-y PMC7434776

[bib54] Wang G., Xie M., Wu W. et al. (2021). Structures and functional diversities of ASFV proteins. Viruses 13, 2124. 34834930 10.3390/v13112124 PMC8619059

[bib55] Wang N., Zhao D., Wang J. et al. (2019). Architecture of African swine fever virus and implications for viral assembly. Science 366, 640–644. 31624094 10.1126/science.aaz1439

