# Supplementary material for: African swine fever virus DEAD-box helicase D1133L promotes OGG1-driven incision of genomic 8-oxoG via HDAC5 deacetylation
Source: J Mol Cell Biol. 2025 Aug 27;17(7):mjaf029. doi: 10.1093/jmcb/mjaf029 (PMC12902152; doi:10.1093/jmcb/mjaf029)
Supplement: mjaf029_Supplemental_File [file mjaf029_supplemental_file.pdf]

Supplementary material

Supplementary Material and methods

Plasmid construction

Plasmids encoding HA-OGG1, Flag-D1133L, Flag-NT-D1133L, Flag-CT-D1133, Myc-HDAC5, Myc-p300 and Myc-CBP were constructed by inserting the synthesized sequences into pCDNA3.1 with indication tags fused to the 3′ end. The construction of these expression vectors was performed by Sangon Biotech, China.

Knockout of OGG1

The establishment of OGG1 knockout cell lines refers to previous study with some modifications (Visnes *et al.*, 2018). OGG1-depletion were performed by infection of MA104 cells with lentivirus expressing sgRNA. The sequences of sgRNAs targeting OGG1 (5′-GATGCGGGCGATGTTGTTGTTGG-3′ and 5′-AACAACATCGCCCGCATCACTGG-3′) was designed by Tsingke Biotechnology, China. Briefly, the sgRNAs were incorporated into pSpCas9(BB)-2A-Puro plasmid, followed by co-transfected into HEK 293T cells with the packaging plasmids pVSVg (AddGene 8454) and psPAX2 (AddGene 12260) for 6 h at 37℃. Media was replaced with 2.5 mL of fresh high-BSA containing growth DMEM and incubated for 48 h. Then the media containing the lentivirus particles were harvested and used for infection of MA104 cells in 6-well plate in medium containing 5 μg/mL of puromycin for 48 h. Cells were re-seeded into 96-well plates to obtain single colonies. Each expanded clone was tested for OGG1 expression by western blot analysis using OGG1 antibody.

8-oxoG measurement

Genomic DNAs were extracted by using a Blood & Cell Culture DNA Mini Kit (13323, Qiagen, Germany). The level of 8-oxoG was measured by a commercial ELISA kit (4661, Chemicalbook, China) according to the manufacturer's instructions. The absorbance was determined by using a microplate reader (ThermoFisher Scientific, USA) at wavelength of 450 nm.

Western blotting and immunoprecipitation

The treated and control cells were harvested and lysed in RIPA buffer (89900, ThermoFisher Scientific) supplemented with protease inhibitor cocktail (87786, ThermoFisher Scientific) for 3 h on ice. Then the whole protein extracts were pelleted and separated by SDS-PAGE and transferred to

polyvinylidene difluoride membrane (10600023, Cytiva), followed by blocking with 5% (w/v) skimmed milk for 1 h at room temperature (RT). Next, the membrane was incubated with the corresponding primary and HRP-conjugated secondary antibodies. The levels of protein were detected and quantified by Quantity One software (Bio-Rad Laboratories, USA).

For immunoprecipitation, cells were collected and lysed in IP lysis buffer (87787, ThermoFisher Scientific) supplemented with protease inhibitor cocktail for 3 h on ice, then the indicated antibodies were added into the cell lysate and mixed overnight at 4 ℃ followed by the addition of protein A/G magnetic beads (26162, Thermo Scientific). The immunoprecipitants were washed and analyzed by western blotting.

Confocal microscopy

HEK 293 cells or MA104 cells were cultured in confocal dishes, fixed with 4% paraformaldehyde (R37814, ThermoFisher Scientific) in PBS for 10 min at RT followed by PBS washes. Subsequently, cells were permeabilized with 0.1% Triton X-100 (X100, Sigma Aldrich) in PBS for 20 min at RT, followed by blocking with 5% BSA in PBS for 1 h. Next, the cells were incubated with corresponding primary antibodies overnight at 4 ℃ followed by incubation with fluorochrome-conjugated secondary antibodies for 1 h. All images were captured using a TCS SP8 confocal fluorescence microscope (Leica, Germany).

Reverse transcription-coupled quantitative PCR (RT-qPCR)

Total RNA was extracted from the collected samples using TRIzol (15596018, Invitrogen) according to the manufacturer's protocol. Expression analysis of all target genes was conducted using qRT-PCR with a Step Prime Script RT PCR Kit (RR064A, Takara, Japan) following the manufacturer's instructions. The relative amounts of each designated gene were calculated by using the comparative cycle threshold ($2^{-\Delta\Delta CT}$) and normalized to *GAPDH*. All experiments were independently repeated three times.

Cell viability assay

Cell viability assay was performed by the CCK-8 kit (Cat K1018, APExBIO, USA). Cells were seeded in 96-well plates and cultured for 24 h prior to treatment with menadione or C646 for 48 h. Subsequently, 10% CCK-8 (Bimake, B34304) was added in cells to assess cell viability. Absorbance was measured at 450 nm. Cell viability was calculated according to the formula: cell viability rate (%) = [(OD inhibitor – OD blank) / (OD control – OD blank)] × 100%.

Acetylation assay in vitro

Recombinant His-D1133L was incubated with increased amounts of p300 or CBP in 20 μL acetylation buffer (50 mM Tris, pH 8.0, 50 mM NaCl, 0.1 mM EDTA, 100 μg/mL BSA, 10% glycerol, 1 mM DTT, 1 mM PMSF, and 10 mM sodium butyrate) supplemented with 2 mM Acetyl-CoA at 30 ℃ for 1 h. The reaction was terminated by adding 10× Laemmli buffer (S3401, Sigma-Aldrich). The levels of Ac-D1133L and D1133L were assessed using western blotting with anti-acetylated lysine antibody and anti-His antibody.

Deacetylation assay in vitro

To acetylate D1133L, recombinant D1133L was incubated with CBP or p300 in 10 μL acetylation buffer (50 mM Tris, pH 8.0, 50 mM NaCl, 0.1 mM EDTA, 10% glycerol, 1 mM DTT, 1 mM PMSF) in presence of 2 mM acetyl CoA for 1 h at 30 ℃. Different amounts of recombinant HDAC5 or TSA were then added to the reaction mixtures before incubating in 20 μL deacetylation buffer (50 mM Tris–HCl, pH 9.0, 50 mM NaCl, 4 mM $MgCl_2$, 1 mM DTT, 1 mM PMSF, 10% glycerol) at 30 ℃ for another 1 h and resolved by SDS–polyacrylamide gel electrophoresis (PAGE). The acetylation of D1133L was assessed by western blot using anti-acetylated lysine antibody and anti-His antibody.

Purification of Flag-tagged D1133L

Purified protein was prepared according to the manufacturer's protocol. HEK 293T cells were transfected with Flag-tagged full-length D1133L (FL-D1133L) for 24 h then were lysed in IP lysis buff supplemented with protease inhibitor cocktail and/or HDAC inhibitors (1 μM TSA) on ice for 3 h. Insoluble material was removed by centrifugation at 2000× *g* for 30 min at 4 ℃. Subsequently, the lysate was incubated for 3 h at 4 ℃ with anti-Flag magnetic beads (A36797, Invitrogen) on a rocking platform, followed by rinsing. Immunopurified Flag-tagged D1133L was eluted with NP-40 buffer in presence of 1.5 μg/μL Flag-peptide (A36805, ThermoFisher Scientific) followed by incubation for 15 min at RT on a rocking platform. Proteins in eluted fractions were analyzed by SDS–PAGE and stained with 0.1% Coomassie brilliant blue.

8-oxoG incision assay

The complementary oligonucleotide was annealed from the 28-mer (28 oligomer) template strand containing an 8-oxoG lesion at a specific position. The oligonucleotide sequences are: 5'-GAA CGA CTG T (8-oxoG) A CTT GAC TGC TAC TGA T-3'- Cy5, and 5'- ATC AGT AGC AGT CAA GTC ACA GTC GTT C-3' as previously described (Duan *et al.*, 2020) .

The 8-oxoG incision assay was performed as previously described with some modifications (Stevnsner *et al.*, 2002; Stuart *et al.*, 2004). Various amounts of recombinant His-D1133L and/or

OGG1 protein and pre-annealed DNA substrate (50 nM) were incubated in a 20 μL reaction mixture containing 70 mM HEPES–KOH, 1 mM EDTA, 1 mM DTT, 75 mM NaCl, 5 mM $MgCl_2$, 10% glycerol and 100 g/mL BSA at 37℃ for 30 min. The reaction was stopped by adding formamide loading buffer (80% formamide, 10 mM EDTA, 1 mg/mL xylene cyanol FF, and 1 mg/mL Bromophenol Blue) and heating at 90˚C for 5 min followed by cooling on ice. The samples were analyzed in a denaturing 20% polyacrylamide gel containing 7 M urea and measured by ChemiDoc Imaging Systems (Bio-Rad). Bands were visualized using a ChemiDoc Imaging System and analyzed using ImageJ software (USA).

For 8-oxoG incision assay with immunopurified Flag-tagged D1133L, 50 nM annealed oligonucleotide was incubated with purified proteins and/or OGG1 in reaction buffer containing 20 mM HEPES–KOH, 5 mM EDTA, 5 mM DTT, 75 mM KCl, 5 mM $MgCl_2$, 5% glycerol and 100 μg/mL BSA at 37°C for 4 h. Following the incubation 1 μL 20 mg/mL Proteinase K (P9460, Solarbio) and 1 μL 10% SDS were added to the reaction mixture and incubated at 55℃ for 30 min. Subsequently, glycogen (20 μg), ammonium acetate (8 μL) and ethanol (60 μL) were added to the reaction samples and incubated at –80℃ overnight to precipitate the DNA products and substrates. The precipitated DNAs were then pelleted, dried and boiled in formamide loading buffer as above. The resulting DNA precipitates were analyzed by electrophoresis on 20% polyacrylamide/7 M urea gel in 1× TBE buffer. Bands were visualized using a ChemiDoc Imaging System and analyzed using ImageJ software (USA). Incision activity was determined by calculating the intensity of product bands relative to the combined intensities of substrate and product bands.

Supplementary References

Visnes, T., Cazares-Korner, A., Hao, W., et al. (2018). Small-molecule inhibitor of OGG1 suppresses proinflammatory gene expression and inflammation. Science, *362*, 834-839.

Duan, S., Han, X., Akbari, M., et al. (2020). Interaction between RECQL4 and OGG1 promotes repair of oxidative base lesion 8-oxoG and is regulated by SIRT1 deacetylase. Nucleic Acids Research, *48*, 6530-6546.

Stevnsner, T., Nyaga, S., de Souza-Pinto, N.C., et al. (2002). Mitochondrial repair of 8-oxoguanine is deficient in Cockayne syndrome group B. Oncogene, *21*, 8675-8682.

Stuart, J.A., Hashiguchi, K., Wilson, D.R., et al. (2004). DNA base excision repair activities and pathway function in mitochondrial and cellular lysates from cells lacking mitochondrial DNA. Nucleic Acids Research, *32*, 2181-2192.

Supplementary Figures

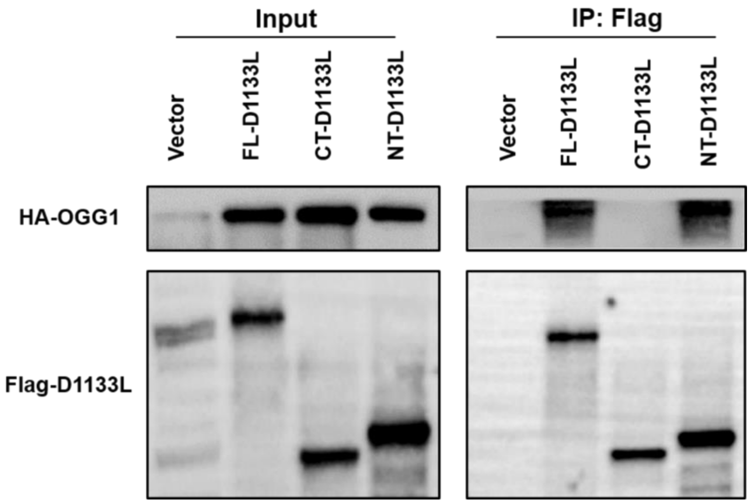

Supplementary Figure S1. D1133L DEAD-box selectively interacts with OGG1.

N-terminal domain (DEAD-box) of D1133L interacts with OGG1 in HEK 293T cells. HA-OGG1 (2 μg) and FL-D1133L, NT-D1133L or CT-D1133L (2 μg each) were co-transfected into HEK 293T cells for 24 hours. Then the interaction was tested by Co-IP.

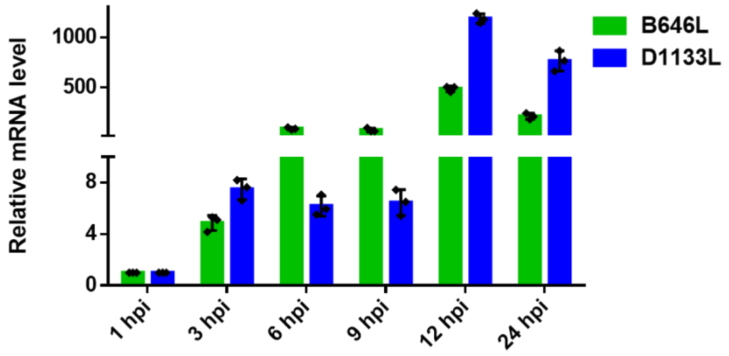

Supplementary Figure S2. The mRNA level of D1133L post ASFV infection.

RT-qPCR was used to determine the mRNA level of *D1133L* and *B646L* in PAM cells post ASFV infection at different time points.

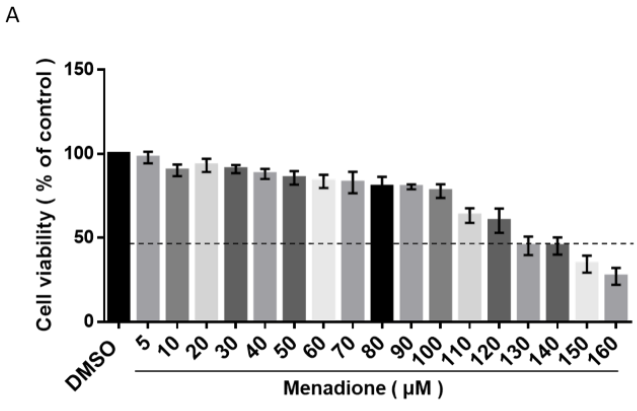

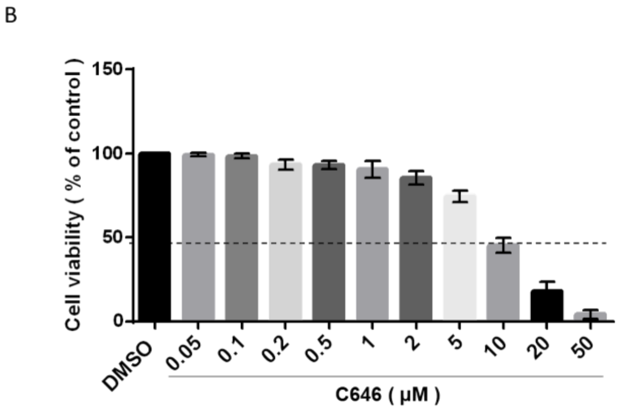

Supplementary Figure S3. Cytotoxicity analysis of menadione or C646 in HEK 293T cells.
Cell viability was determined by using the CCK-8 Kit, and the data were analyzed by using GraphPad Prism.

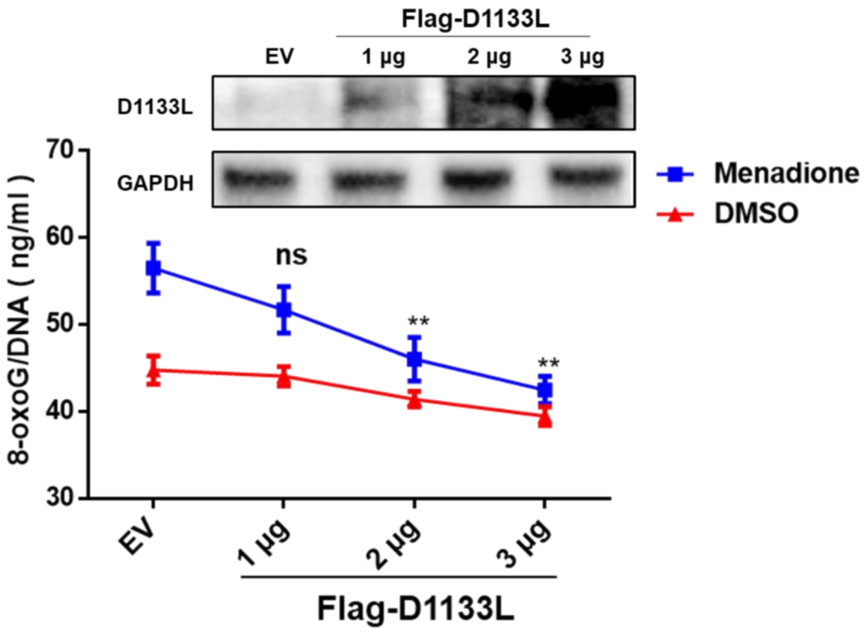

Supplementary Figure S4. The concentration of 8-oxoG was measured by ELISA assay after Flag-D1133L transfection and menadione treatment.
Porcine alveolar macrophage 3D4/21 cells were transfected with Flag-D1133L (0, 1, 2, 3 μg) for 24 hours, then treated with menadione (40 μM) or DMSO for 6 hours. Western blot assay indicated the overexpression of Flag-D1133L. The significance was compared to EV in menadione group, Data are shown as mean ± SD (*n* = 3). **P* < 0.05, ***P* < 0.01; ns, not significant (*t*-test).

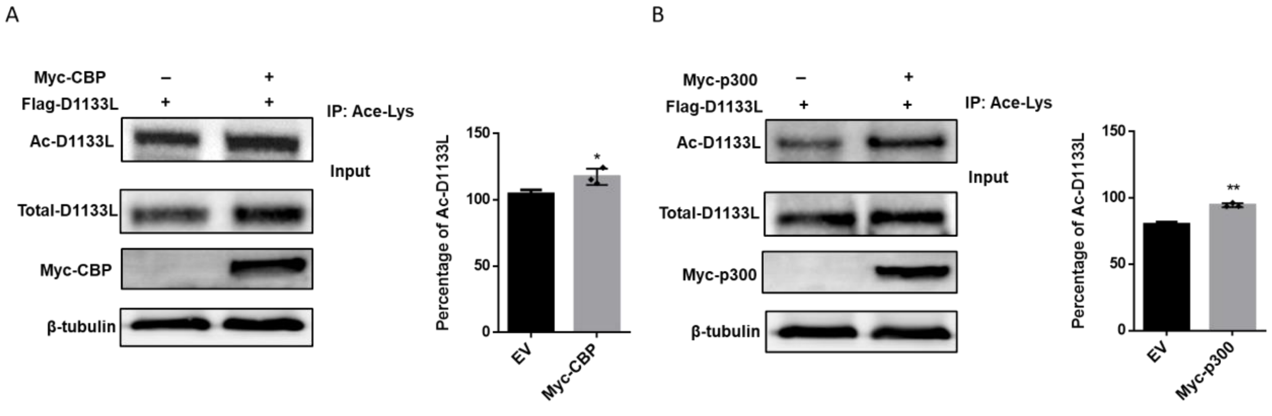

Supplementary Figure S5. D1133L is a substrate for CBP/p300.

Acetylation of D1133L by CBP/p300 in porcine alveolar macrophage 3D4/21. 3D4/21 cells were co-transfected with Flag-D1133L (2 μg), Myc-p300 (2 μg) or Myc-CBP (2 μg) for 24 hours. Acetylated D1133L (Ac-D1133L) was immunoprecipitated using anti-acetylated-lysine antibody and changes were assessed by Western blotting with Flag antibody. The graph on the right shows quantification of relative acetylated D1133L normalized to total D1133L. Data are presented as mean ± SD ($n = 3$). *$P < 0.05$, **$P < 0.01$ (*t*-test).

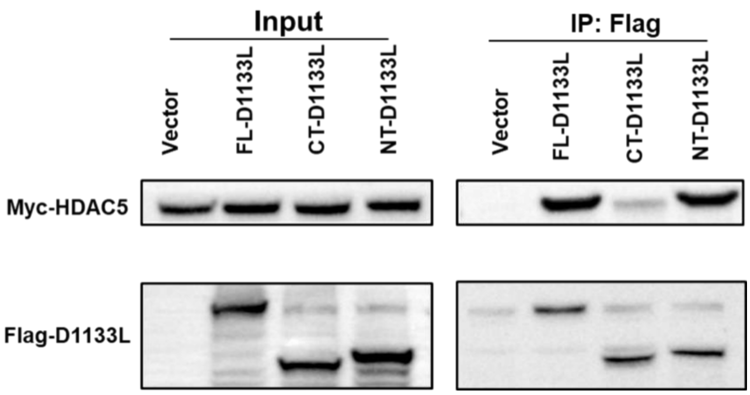

Supplementary Figure S6. N-terminal domain of D1133L interacts with HDAC5.

Immunoprecipitation was performed in HEK 293T cells expressing vector alone or FL-D1133L, NT-D1133L, CT-D1133L (2 μg each) with magnetic beads and analyzed by IB with anti-Flag and anti-Myc antibodies.

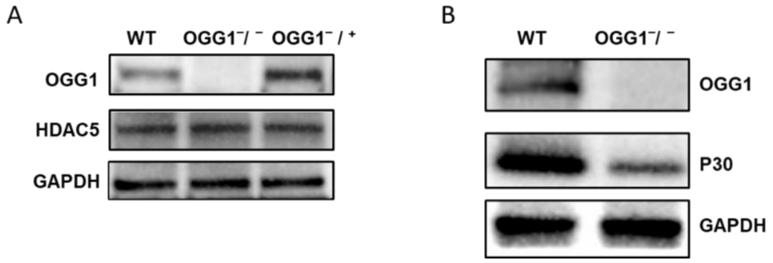

Supplementary Figure S7. The effect of OGG1 depletion on HDAC5 level and ASFV replication. (**A**) Effect of OGG1 knockout and rescue on HDAC5 expression. OGG1 and HDAC5 level were tested by Western blotting in WT, OGG1 knockout ($OGG1^{-}/^{-}$), and OGG1 rescue ($OGG1^{-}/^{+}$) MA104 cells. (**B**) Effect of OGG1 knockout on ASFV replication. WT and OGG1 knockout MA104 cells were infected with ASFV (MOI = 1) for 24 hours, then the level of OGG1 and ASFV-p30 was detected by using Western blotting.

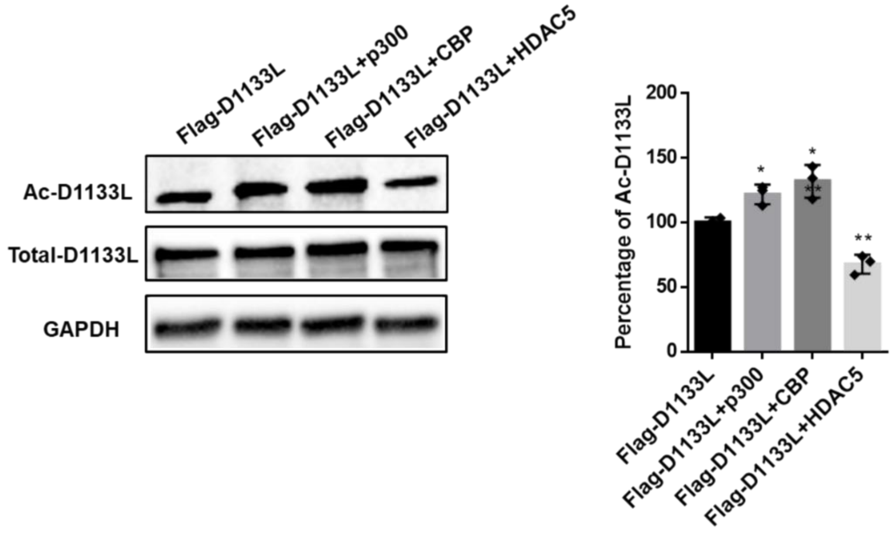

Supplementary Figure S8. The acetylation level of purified Flag-D1133L.

HEK 293T cells were transfected with FL-D1133L (2 μg) alone or co-transfected with CBP/p300 (2 μg), HDAC5 (2 μg) for 24 hours. The cells were then lysed in IP lysis buffer and incubated with anti-Flag magnetic beads. Immunopurified Flag-D1133L was eluted with Flag peptide, and the purified proteins in the eluted fractions were analyzed by Western blotting using anti-Flag and anti-acetylated-lysine antibodies. The graph on the right shows the quantification of relative acetylated D1133L normalized to total D1133L. Data are presented as mean ± SD (n = 3). *$P < 0.05$, **$P < 0.01$ (t-test).

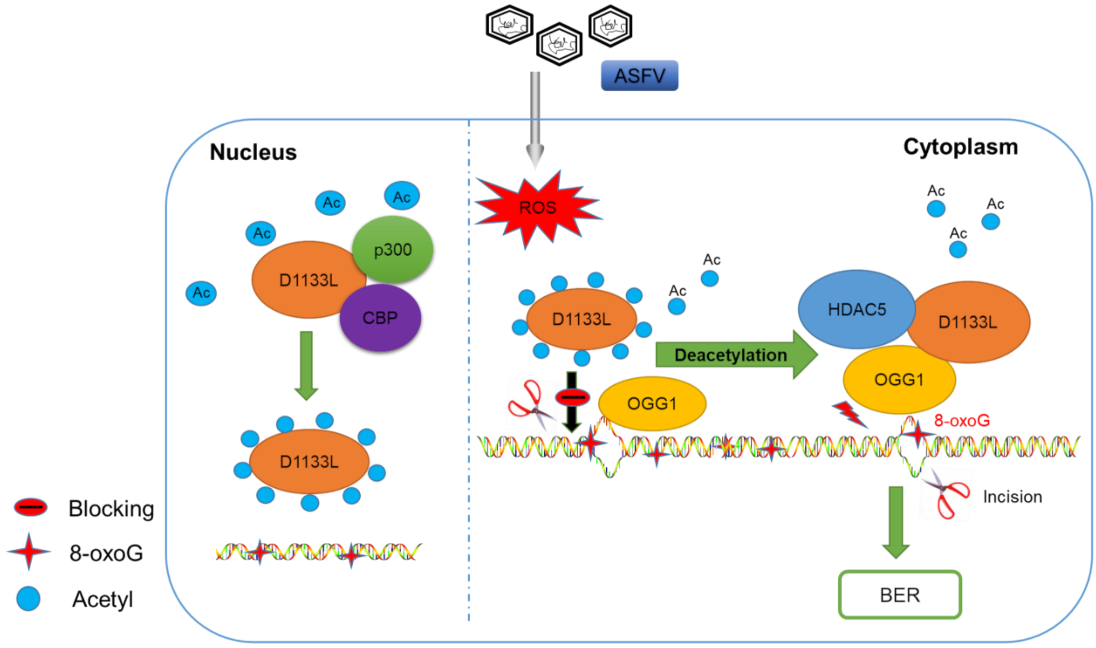

Supplementary Figure S9. Scheme of ASFV DEAD-box helicase D1133L interaction with OGG1 and HDAC5 to facilitate incision of 8-oxoG.

Under ASFV-induced oxidative stress, DEAD-box helicase D1133L undergoes acetylation by CBP/p300 in the nucleus, keeping in a hyperacetylated state. The hyperacetylation of D1133L by host acetyltransferases inhibits its activity in OGG1-mediated 8-oxoG incision. In the cytoplasm, OGG1 interacts with D1133L and HDAC5 to deacetylate D1133L. This process enhances the incision activity of OGG1, contributing to 8-oxoG removal through DNA base excision repair pathway.
